# High-Yield Synthesis of Enantiopure 1,2-Amino Alcohols from l-Phenylalanine via Linear and Divergent Enzymatic Cascades

**DOI:** 10.1021/acs.oprd.1c00490

**Published:** 2022-03-28

**Authors:** Maria L. Corrado, Tanja Knaus, Ulrich Schwaneberg, Francesco G. Mutti

**Affiliations:** † Van’t Hoff Institute for Molecular Sciences, HIMS-Biocat, University of Amsterdam, Science Park 904, Amsterdam 1098 XH, The Netherlands; ‡ Institute of Biotechnology, RWTH Aachen University, Worringerweg 3, Aachen 52074, Germany

**Keywords:** biocatalysis, biocatalytic cascades, amine dehydrogenases, transaminases, alcohol dehydrogenases, alcohol oxidases, phenylethanolamine, 2-phenylglycinol

## Abstract

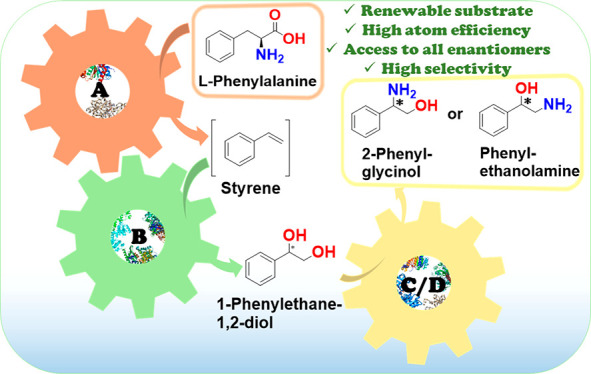

Enantiomerically pure 1,2-amino alcohols are important compounds due to their biological activities and wide applications in chemical synthesis. In this work, we present two multienzyme pathways for the conversion of l-phenylalanine into either 2-phenylglycinol or phenylethanolamine in the enantiomerically pure form. Both pathways start with the two-pot sequential four-step conversion of l-phenylalanine into styrene via subsequent deamination, decarboxylation, enantioselective epoxidation, and enantioselective hydrolysis. For instance, after optimization, the multienzyme process could convert 507 mg of l-phenylalanine into (*R*)-1-phenyl-1,2-diol in an overall isolated yield of 75% and >99% ee. The opposite enantiomer, (*S*)-1-phenyl-1,2-diol, was also obtained in a 70% yield and 98–99% ee following the same approach. At this stage, two divergent routes were developed to convert the chiral diols into either 2-phenylglycinol or phenylethanolamine. The former route consisted of a one-pot concurrent interconnected two-step cascade in which the diol intermediate was oxidized to 2-hydroxy-acetophenone by an alcohol dehydrogenase and then aminated by a transaminase to give enantiomerically pure 2-phenylglycinol. Notably, the addition of an alanine dehydrogenase enabled the connection of the two steps and made the overall process redox-self-sufficient. Thus, (*S*)-phenylglycinol was isolated in an 81% yield and >99.4% ee starting from ca. 100 mg of the diol intermediate. The second route consisted of a one-pot concurrent two-step cascade in which the oxidative and reductive steps were not interconnected. In this case, the diol intermediate was oxidized to either (*S*)- or (*R*)-2-hydroxy-2-phenylacetaldehyde by an alcohol oxidase and then aminated by an amine dehydrogenase to give the enantiomerically pure phenylethanolamine. The addition of a formate dehydrogenase and sodium formate was required to provide the reducing equivalents for the reductive amination step. Thus, (*R*)-phenylethanolamine was isolated in a 92% yield and >99.9% ee starting from ca. 100 mg of the diol intermediate. In summary, l-phenylalanine was converted into enantiomerically pure 2-phenylglycinol and phenylethanolamine in overall yields of 61% and 69%, respectively. This work exemplifies how linear and divergent enzyme cascades can enable the synthesis of high-value chiral molecules such as amino alcohols from a renewable material such as l-phenylalanine with high atom economy and improved sustainability.

## 1 Introduction

**Figure 1:**
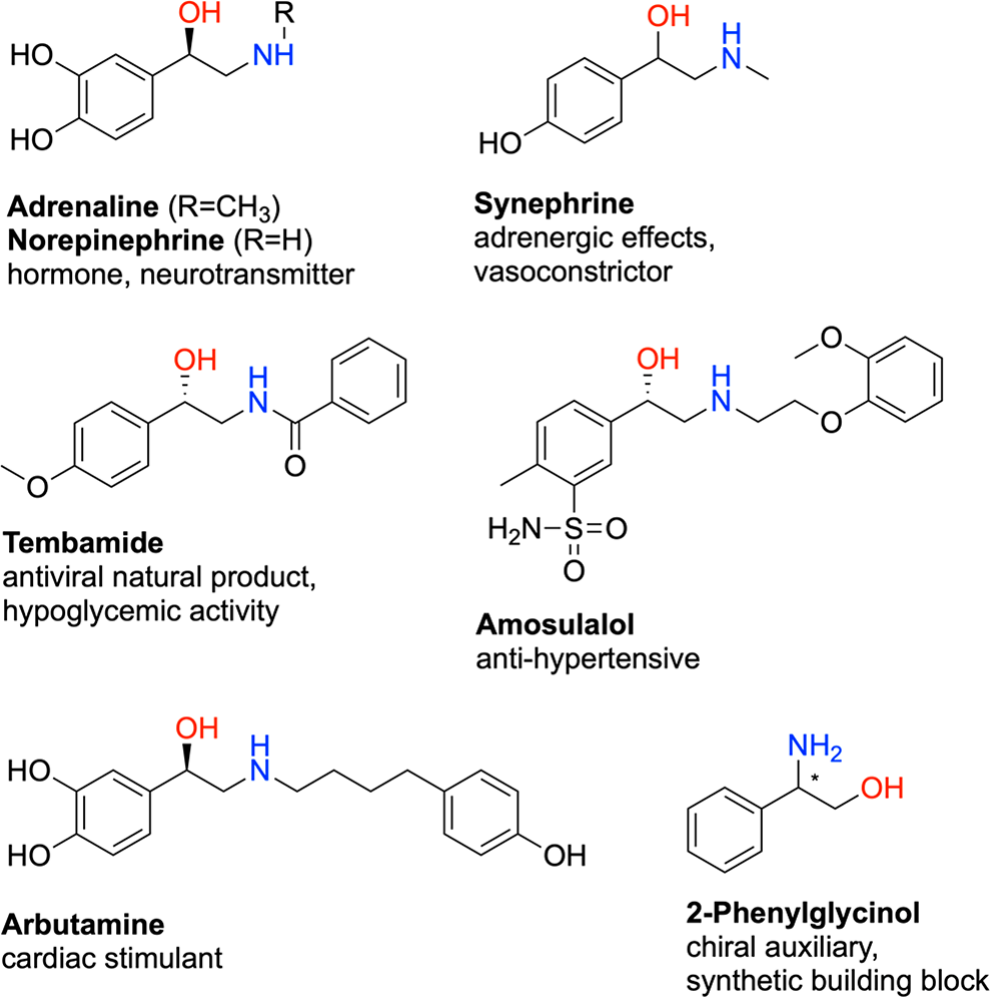
Examples of biologically active compounds and chiral auxiliaries bearing phenylethanolamine or 2-phenylglycinol moieties.

Over the past two decades, biocatalysis has made a major contribution toward sustainable chemical synthesis, in particular for the highly selective syntheses of high-value chiral molecules.^1−10^ In this context, the utilization of biocatalysis to convert biomass-derived starting materials into chiral molecules as intermediates or final products for the manufacture of active pharmaceutical ingredients, flavors, fragrances, agrochemicals, and fine chemicals can make a decisive contribution to enabling a circular economy.^11−18^ Natural amino acids are abundant and inexpensive biobased feedstocks produced by fermentation that have been marginally used as starting materials for the chemical synthesis of chiral molecules.^19−21^ For instance, the fermentative production of l-phenylalanine with a titer above 70 g per liter of culture can be accomplished from glucose or glycerol using *Escherichia coli* strains in which the l-phenylalanine biosynthesis pathway (i.e., the shikimate pathway) has been engineered.^22−26^ In contrast, chiral 1,2-amino alcohol motifs are widespread in biologically active compounds and bioactive natural products such as antibiotics, neurotransmitters, β-adrenergic blockers, and antiviral drugs.^27−29^ They also find application in asymmetric organic synthesis as ligands, chiral auxiliaries, and even organocatalysts.^30−32^ As illustrated in Figure 1, chiral phenylethanolamines and 2-phenylglycinols are particularly important in this context for their chemical and biological properties.

Highly selective asymmetric synthesis methods remain widely sought after to obtain these and many other valuable compounds and intermediates thereof in high chemical and optical purities. Synthetically applied methods comprise the Sharpless asymmetric aminohydroxylation of terminal olefins,^33,34^ the asymmetric hydrogenation of prochiral amino ketones,^35^ and the ring-opening of an epoxide with an amine as the nucleophile.^36−39^ Nevertheless, these methods have some drawbacks related to their selectivity and sustainability, as the phenylethanolamines are hardly ever obtained in their enantiomerically pure forms and toxic metals and reagents are often required in superstoichiometric amounts.^40−42^ Consequently, resolution techniques are still mainly used for the preparation of optically pure 1,2-amino alcohols.^35^ Notably, biocatalytic strategies for the synthesis of chiral 1,2-amino alcohols have also been developed;^43−52^ however, only a few methods are currently available for the specific synthesis of chiral phenylethanolamines and 2-phenylglycinols.^44,49,53,54^

A chemo-biocatalytic route toward phenylethanolamines entails the ring-opening of styrene oxide with ammonia under microwave irradiation; styrene oxide is obtained from the bioepoxidation of styrene with a styrene monooxygenase.^55^ In full biocatalytic approaches, linear cascades have been demonstrated to be viable options due to their often high selectivity and atom efficiency.^56,57^ Notable examples are a multienzymatic cascade for the asymmetric synthesis of (*R*)-2-phenylglycinol ((*R*)-**7**) from racemic styrene oxide (**4**);^58^ a one-pot three-step enzymatic process to convert a series of halo ketones to the corresponding amino alcohols, including the natural antiviral product (*S*)-tembamide;^29^ the conversion of styrene to (*S*)-phenylethanolamine ((*S*)-**9**) by modular cascade biocatalysis;^59^ and the engineered hemoprotein-catalyzed direct enantioselective aminohydroxylation of olefins.^28^

In this work, we exemplify the potential of biocatalysis for the synthesis of high-value aromatic 1,2-amino alcohols such as optically active phenylethanolamines and 2-phenylglycinols through enzymatic cascade reactions starting from l-phenylalanine as a renewable material. These cascades harness some of the engineered enzymes and reactions that our group has developed over the past five years.

## 2 Results and Discussion

### 2.1 Conversion of l-Phenylalanine into (*R*)-1-Phenylethane-1,2-diol

**Scheme 1:**
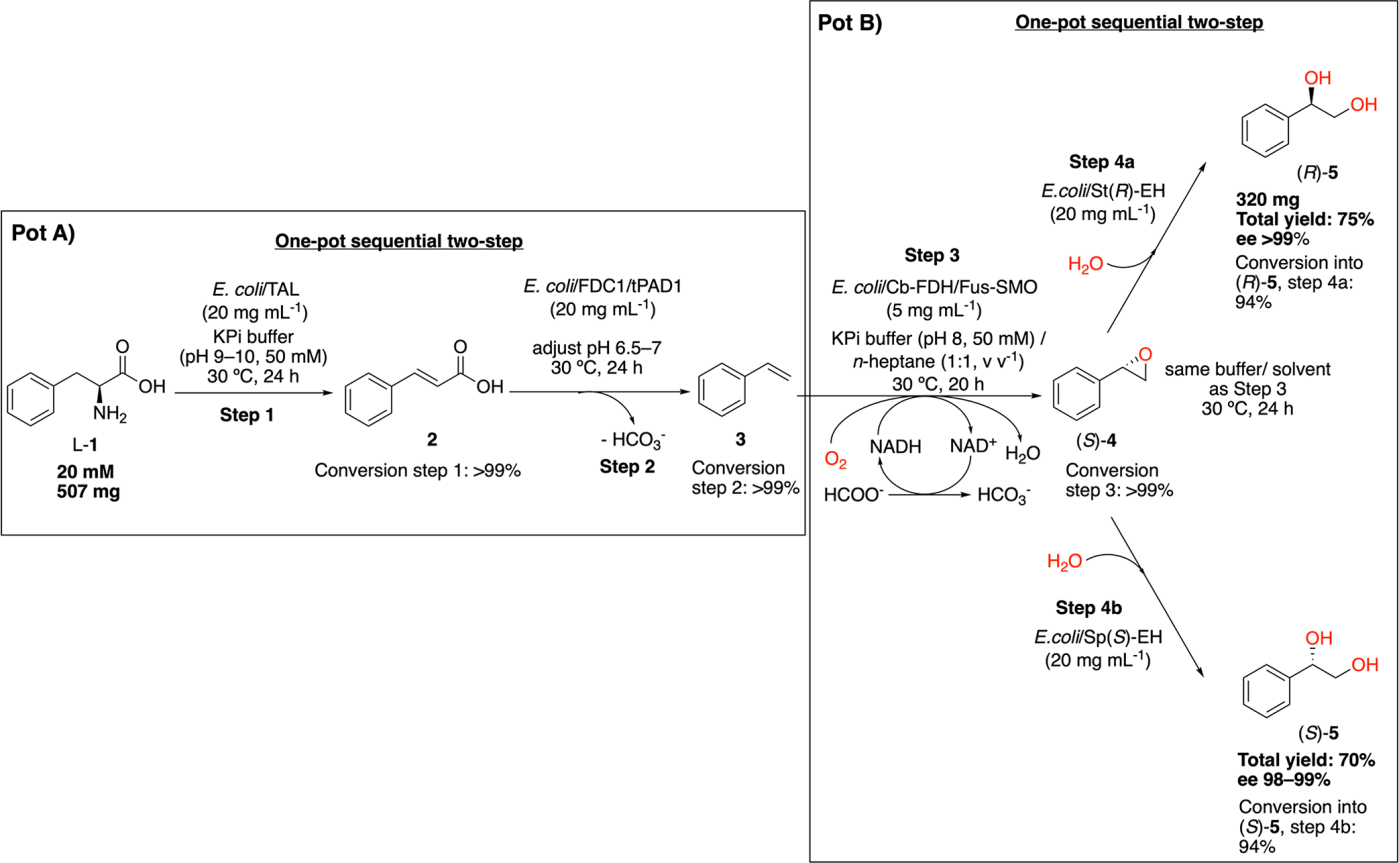
Two-Pot Four-Step Sequential Biocatalytic Cascades for the Conversion of l-Phenylalanine (l-**1**) into (*R*)- or (*S*)-1-Phenylethane-1,2-diol ((*R*)- or (S)-**5**) There is only one intermediate extraction work-up after step 2.

Scheme 1 depicts the first part of our synthetic strategy in which l-phenylalanine (l-**1**) is converted into (*R*)-1-phenylethane-1,2-diol ((*R*)-**5**) through two-pot four-step sequential biocatalytic cascades (or two-pot four-stage cascades, as all four steps are separated in time) with only one intermediate extraction step. l-**1** (20 mM, 3.1 mmol, 507 mg) was first deaminated to cinnamic acid (**2**) with >99% conversion and >99% chemoselectivity (see Figure S2). The reaction was catalyzed by lyophilized *E. coli* cells (3 g, 20 mg mL^–1^) expressing a tyrosine ammonia lyase from *Rhodobacter sphaeroides* (TAL),^60^ which were suspended in a KPi buffer (pH 9, 50 mM, 150 mL) for 24 h at 30 °C. Following the removal of the cell pellets by centrifugation, the reaction mixture from step 1 was directly reacted in the subsequent step. The pH of the mixture was lowered to 6.5–7 via the addition of HCl, and lyophilized *E. coli* cells (3 g, 20 mg mL^–1^) expressing a ferulic acid decarboxylase from *Saccharomyces cerevisiae* (FDC1/tPAD1)^61^ were added to perform the decarboxylation of **2** to styrene (**3**) over 24 h at 30 °C. At the end of the reaction (conversion step 2 > 99%, see Figure S3), **3** was extracted with *n*-heptane and used directly in the next one-pot two-step (or one-pot two-stage) sequential cascade. The first step of the second pot (Scheme 1, pot B) was a biocatalytic epoxidation performed in an organic/aqueous biphasic system. The influence of the ratio of the two phases on the conversion was initially tested on an analytical scale (total volume of 1 mL), which showed that a 1:1 volume ratio was the optimal condition (see SI section 3.1). Therefore, the solution of *n*-heptane (150 mL) containing intermediate **3** was combined with a KPi buffer (pH 8, 50 mM, 150 mL) containing lyophilized *E. coli* cells (5 mg mL^–1^, related to the aqueous phase) expressing our previously reported chimeric styrene monooxygenase (Fus-SMO), where the reductive (StyB) and monooxygenase (StyA) enzyme units were fused together using a flexible linker.^62^ This biocatalytic epoxidation step required reducing equivalents that were provided by a catalytic amount of the reduced nicotinamide adenine dinucleotide coenzyme (NADH), which was generated in situ and recycled from NAD^+^ (1 mM) by a formate dehydrogenase (Cb-FDH) and sodium formate (100 mM, 5 equiv). Notably, Fus-SMO and Cb-FDH were produced together in the same *E. coli* cells through cloning and the balanced expression of both genes in a Duet-vector. This is an improvement on previous work in which the enzymes were coexpressed using different plasmids in the same host.^62^ After a 20 hr reaction time at 30 °C (>99% conversion and perfect chemoselectivity), the same reaction pot containing the (*S*)-styrene epoxide ((*S*)**-4**) product was subjected to the next step, where lyophilized *E. coli* cells (3 g, 20 mg mL^–1^) expressing an epoxide hydrolase from *Solanum tuberosum* St(R)-EH were simply added to the mixture.^63−65^ The regioselective biocatalytic hydrolysis step was run for an additional 24 h and proceeded with the full inversion of the stereochemical configuration of (*S*)**-4** to yield (*R*)-1-phenylethane-1,2-diol ((*R*)-**5**) in a quantitative conversion and 94% chemoselectivity. The product was recovered by separation between the *n*-heptane phase and the aqueous phase, the latter of which was further extracted with methyl-*tert*-butyl ether. The organic phases were then dried with anhydrous MgSO_4_, and the solvent was removed. In summary, at the end of this two-pot four-step sequential biocatalytic process and workup (see the Experimental Part for details), (*R*)-**5** was recovered from l-**1** in a 75% overall isolated yield (320 mg) with high chemical (>99%) and optical (ee > 99%) purities. Notably, intermediate purification steps were not required, thereby minimizing waste generation and work time. (*S*)-Configured 1-phenylethane-1,2-diol ((*S*)-**5**) could also be generated (98–99% ee, 70% yield, 240 mg from (*S*)**-4**) in a similar manner under not-fully optimized conditions by changing the selectivity of the epoxide hydrolase in the last hydrolytic step (see SI section 3.2). We performed this reaction using an epoxide hydrolase from *Sphingomonas* sp. HXN200 (Sp(*S*)-EH).^63−65^

Products (*R*)-**5** and (*S*)-**5** were used as starting materials in two subsequent and distinct one-pot biocatalytic cascades for the synthesis of either optically pure 2-phenylglycinol (**7**) or phenylethanolamine (**9**), as described in the following sections.

### 2.2 Conversion of (*R*)-1-Phenylethane-1,2-diol into (*S*)- and (*R*)-2-Phenylglycinol

**Scheme 2:**
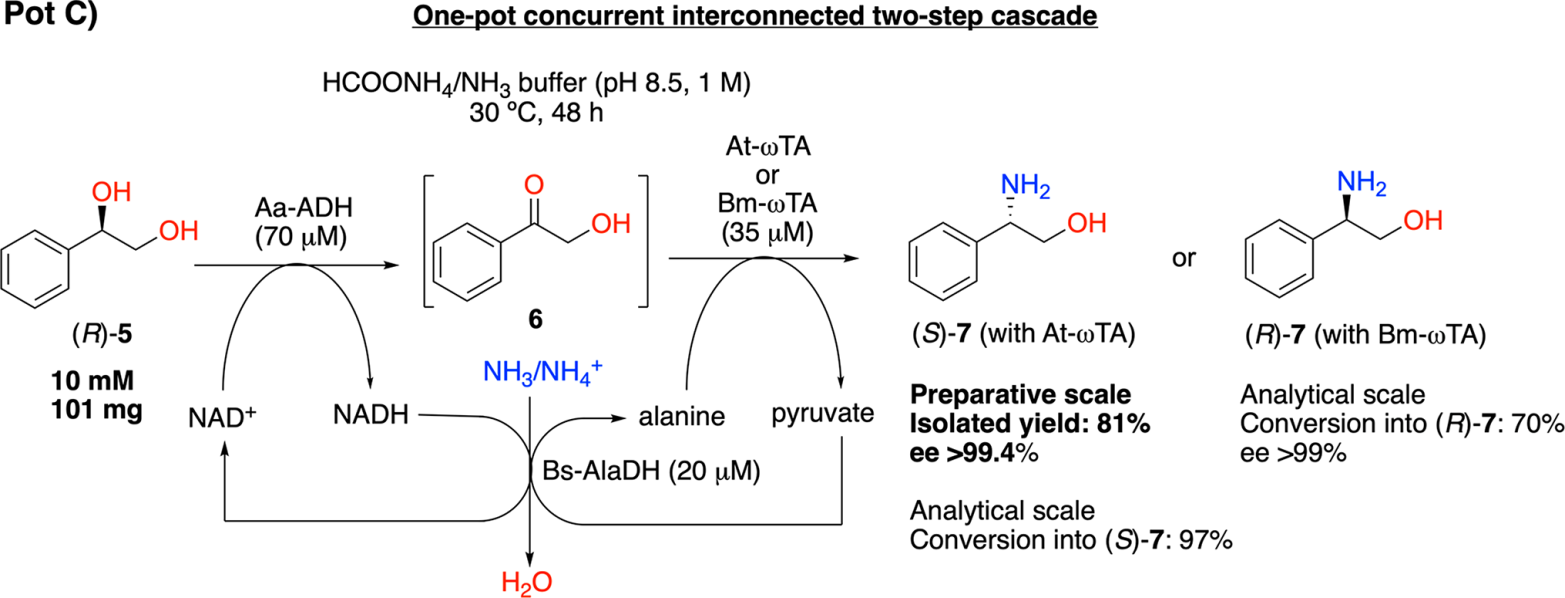
One-Pot Concurrent Interconnected Two-Step Biocatalytic Cascade for the Conversion of (*R*)-1-Phenylethane-1,2-diol ((*R*)-**5**)) into (*R*)- or (*S*)-Phenylglycinol ((*R*)- or (*S*)-**7**))

At this stage, we initially intended to convert (*R*)- or (*S*)-**5**, obtained as previously reported, into (*R*)- or (*S*)-2-phenylglycinol ((*R*)- or (*S*)-**7**) through the one-pot combination of a “secondary” NAD-dependent alcohol dehydrogenase (ADH) and an amine dehydrogenase (AmDH), thus following our previously developed strategy for the synthesis of optically pure phenylpropanolamines.^63,66^ However, all the tested AmDHs proved to be unsuitable for this process (data not shown). Therefore, we turned our attention to the alcohol amination by combining an ADH with an ω-transaminase (ωTA), thus following our alternative strategy for the synthesis of phenylpropanolamines (Scheme 2).^64,67^

In this alcohol amination cascade, Aa-ADH oxidizes (*R*)-**5** to the hydroxyketone intermediate (**6**) and then the ωTA performs the transamination of the carbonyl moiety to yield either (*R*)-**7** or (*S*)-**7**; additionally, the NAD^+^ coenzyme and alanine as the amine donor are internally recycled from NADH and pyruvate, respectively, by an alanine dehydrogenase from *Bacillus sphaericus* (Bs-AlaDH)^68^ at the expense of the ammonia and ammonium species that are provided by the reaction buffer.

The optimal biocatalysts for this transformation were initially tested for the separated reactions on an analytical scale. First, we investigated the oxidation of commercially available *rac*-**5** (20 mM) to the hydroxyketone (**6**) in a Tris-HCl buffer (pH 7.5, 50 mM; final reaction volume of 1 mL) using 12 different alcohol dehydrogenases, namely Bs-BDHA,^69,70^ Pp-ADH,^71,72^ Sy-ADH,^73^ Rs-ADH,^74^ Ls-ADH,^75^ three variants of Te-ADH (v1, v2, and v3),^76^ Aa-ADH,^77^ Lbv-ADH,^78^ and Lb-ADH.^79^ For details on the abbreviations of enzyme names and related preparations, see SI section 1 and Table S1. The reaction with Ls-ADH was carried out in KPi buffer at pH 6.5 (100 mM) rather than pH 7.5 because previous tests performed in our laboratory demonstrated that this enzyme was more sensitive toward higher pH values. The tests were conducted in the presence of either NAD^+^ or NADP^+^ (1 mM) depending on the selectivity of the ADH. NAD^+^ and NADP^+^ were internally recycled with specific oxidoreductases (10 μM), which consume molecular oxygen as oxidant. NOx from *Streptococcus mutans*(80) and YcnD from *Bacillus subtilis*(81) were used for the reoxidation of NADH and NADPH, respectively. High conversions into **6** were observed when Aa-ADH, Lbv-ADH, Bs-BDHA, and Lb-ADH were used (considering both that the substrate **5** was used as racemate and that these ADHs have a preference toward one of the two enantiomers). Moderate conversions were also observed with Ls-ADH and Rs-ADH, while the remaining ADHs were not active toward the target substrate (SI section 4 and Table S3). Among this latter group, Sy-ADH, Pp-ADH, and Te-ADH-v3 are described as “non-stereoselective” ADHs. Since these enzymes were found not to be catalytically active toward **5**, the utilization of *rac*-**5** as a possible intermediate was ruled out at this stage. However, this is not a synthetic limitation since optically pure **5** can be efficiently obtained via the enzymatic strategy illustrated in this work. Therefore, we tested the best-performing ADH from the previous set of experiments with either enantiopure (*R*)-**5** or enantiopure (*S*)-**5** (10 mM, SI section 4 and Table S4). Among these four best ADHs, we excluded Lb-ADH because it was NADP-dependent. In fact, the use of a NAD-dependent ADH is more suitable for our intended final cascades, and NAD^+^ is also cheaper than NADP^+^. Lbv-ADH converted (*S*)-**5** into **6** with >99% conversion, whereas Aa-ADH and Bs-BDHA converted (*R*)-**5** into **6** with 84% and 69% conversion, respectively (SI section 4 and Table S4).

In the next step, we investigated the cascade from (*R*)- or (*S*)-**5** to (*R*)- or (*S*)-**7** using combinations of the three previously selected ADHs (i.e., Aa-ADH, LBv-ADH, and Bs-BDHA) and five stereocomplementary ω-transaminases, namely At-ωTA,^82−84^ Cv-ωTA,^85^ Bm-ωTA,^86^ Ac-ωTA,^87,88^ and Vf-ωTA.^89,90^ For details on the abbreviations of enzyme names, see SI section 1 and Table S1. The reactions were carried out at 30 °C for 48 h in a HCOONH_4_ buffer (pH 8.5, 1 M) supplemented with NAD^+^ (1 mM), d- or l-alanine (50 mM), ω-TA (varied concentrations of 35–60 μM), ADH (varied concentrations of 24–70 μM), Bs-AlaDH (20 μM), and substrate (10 mM). The experiments showed the inherently lower activity of Cv-ωTA toward the in situ generated intermediate **6** compared with those of At-ωTA and Bm-ωTA. Furthermore, selected experiments where Ac-ωTA and Vf-ωTA were used did not yield any conversion (see SI section 5 and Tables S5 and S6). Under the optimal reaction conditions (Table 1), both (*S*)-**7** and (*R*)-**7** were obtained from (*R*)-**5** in high yields and excellent optical purities using Aa-ADH from *Aromatoleum aromaticum* combined with At-ωTA from *Aspergillus terreus* and Bm-ωTA from *Bacillus megaterium*.

**Table 1:**
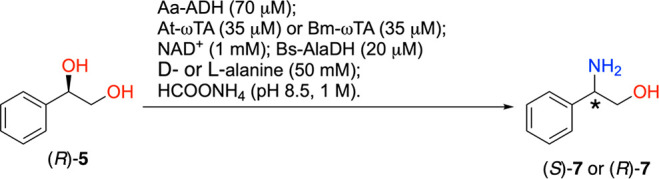
One-Pot Alcohol Amination of (*R*)-**5** (10 mM) to Yield Either (*S*)-**7** or (*R*)-**7**^a^

entry	enzymes	total conv. [%]	conv. into **7** [%]	ee of **7** [%]^b^
1	Aa-ADH/At-ωTA	>99	97 ± < 1	>99 (*S*)
2	Aa-ADH/Bm-ωTA	71 ± < 1	70 ± < 1	>99 (*R*)

a The reaction was catalyzed by Aa-ADH from *Aromatoleum aromaticum* (70 μM), which was combined with either At-ωTA from *Aspergillus terreus* or Bm-ωTA from *Bacillus megaterium* (35 μM) in HCOONH_4_ buffer (pH 8.5, 1 M) at 30 °C for 48 h.

b Determined by RP-HPLC (C18 HD column) following the derivatization of the amino group with GITC. Reactions were performed in duplicate, and results are reported as the average of the two samples.

To prove the synthetic applicability of the one-pot biocatalytic amination, the bioconversion of (*R*)-**5** into (*S*)-**7** was performed on a 101 mg scale. The product (*S*)-**7** was obtained in an 81% isolated yield with 98% purity and >99.4% ee (see the Experimental Part for details).

### 2.3 Conversion of (*S*)- and (*R*)-1-Phenylethane-1,2-diol into (*S*)- and (*R*)-Phenylethanolamine

**Scheme 3:**
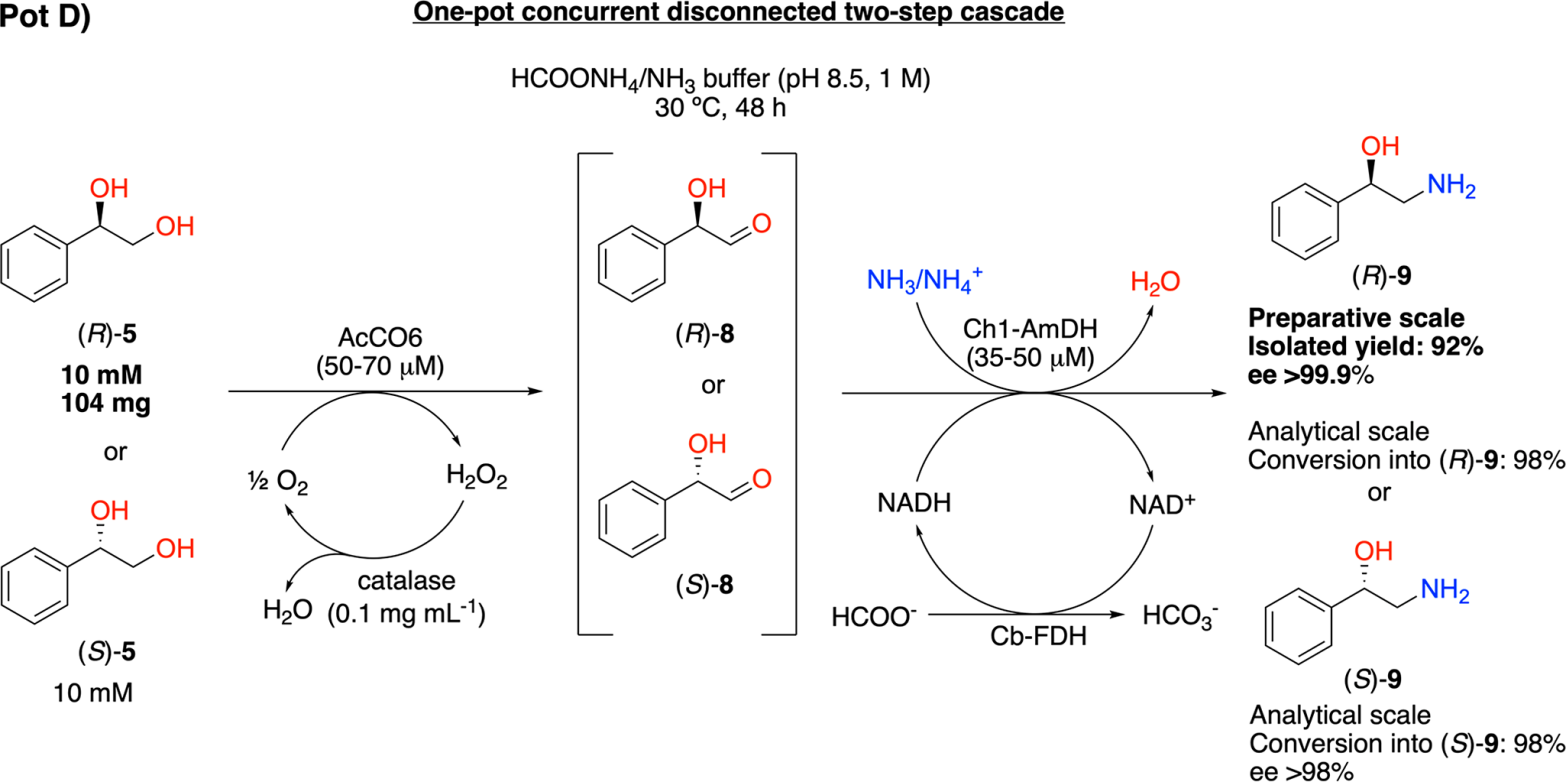
One-Pot Concurrent Disconnected Two-Step Biocatalytic Cascade for the Conversion of (*R*)- or (*S*)-1-Phenylethane-1,2-diol ((*R*)-**5** or (*S*)-**5**) into (*R*)- or (*S*)-Phenylethanolamine ((*R*)- or (*S*)-**9**)

At this stage, we intended to convert (*R*)-**5** or (*S*)-**5**, obtained as previously reported, into either (*R*)- or (*S*)-phenylethanolamine ((*R*)- or (*S*)-**9**) through the one-pot combination of a “primary” ADH and an amine dehydrogenase (AmDH). We initially tested the oxidation of intermediate **5** into 2-hydroxy-2-phenylacetaldehyde (**8**) using an alcohol dehydrogenase. The hT-ADH from *Bacillus stearothermophilus*,^91^ and the HL-ADH from *Equus caballus* (i.e., horse liver)^92^ were tested, as both have been known to oxidize the primary alcohol functionalities of molecules similar to **5**. Ht-ADH was used in its purified form (50 μM), whereas HL-ADH was a commercially available enzyme and was used as the lyophilized cell lysate (2 mg mL^–1^ crude enzyme; activity of 0.52 U mg^–1^; 20% protein content). In both cases, the biocatalytic oxidation of *rac*-**5** (20 mM) was performed in a Tris-HCl buffer (pH 7.5, 50 mM; final reaction volume of 1 mL) in the presence of a NAD^+^ recycling system (1 mM NAD^+^; 10 μM NOx). Contrary to our expectations, hT-ADH exhibited no activity toward substrate *rac*-**5**, while HL-ADH produced the hydroxyketone isomer **6** rather than the desired product **8**, thus actually acting as a secondary ADH. Such activity of HL-ADH on secondary alcohol moieties with certain molecules was also reported in the literature.^93−97^ Therefore, we envisioned an alternative strategy for the amination of the primary alcohol moiety of **5** that combined a variant of the choline oxidase (AcCO6) originated from *Arthrobacter chlorophenolicus*(98) with an AmDH (Ch1-AmDH).^99,100^ Thus, this cascade for the bioamination of the diol **5** is comprised of two concurrent, albeit disconnected, steps that must confer a more favorable thermodynamic equilibrium (Scheme 3).

Another advantage of AcCO6 is that it is an oxidase; therefore, its activity does not depend on the NADH/NAD^+^ coenzyme. However, a catalase must be added to prevent the possible deactivation of any enzyme in the reaction mixture due to the formation of H_2_O_2_ as a side product in the oxidation reaction. Furthermore, the addition of a catalytic amount of NAD^+^ and a formate dehydrogenase from *Candida boidinii* (Cb-FDH) is required in the second reductive amination step of the cascade for the in situ generation and recycling of NADH.^76,100^

The reactions were performed in a HCOONH_4_ buffer (pH 8.5, 1 M) that provided both the source of the amino group and the reducing hydride (i.e., for NADH regeneration) for the reductive amination. The initial set of experiments (SI section 6 and Table S7) were carried out using an equimolar ratio of AcCO6 and Ch1-AmDH (50 μM each) in a HCOONH_4_ buffer (pH 8.5, 1 M; 0.5 mL) supplemented with a catalytic amount of NAD^+^ (1 mM) and a catalase (0.1 mg mL–1). **5** was used as the substrate either as a racemate or as a single enantiomer in the *R*- or *S*-absolute configuration (10 or 20 mM). The quantitative conversion of *rac*-**5** into the target amino alcohol **9** was detected at the 10 mM scale, and oxidation at the 20 mM scale afforded 90% conversion. Due to the racemic form of the substrate, the enantiomeric excess of the product was low in both cases (10% ee (*S*)). Therefore, although AcCO6 did not strongly discriminate between the two enantiomers of **5**, we observed a small preference for the oxidation of (*S*)-**5** over (*R*)-**5**. Next, the same reaction and conditions were investigated to convert optically pure **5** as substrates, obtained from l-phenylalanine (l-**1**) via the first cascades, into chiral products **9**. (*S*)-**5** was converted, resulting in a 98% (at 10 mM substrate concentration) or 88% (at 20 mM substrate concentration) yield of (*S*)-**9**. As expected, the enantiomeric excess of the starting material (*S*)-**5** (98–99% ee) was the same as that in the final product (*S*)-**9**. (*R*)-**5** was also converted into (*R*)-**9**, with 92% or 72% conversion at a substrate concentration of 10 or 20 mM, respectively, and showed the same enantiomeric excess of the starting material (>99% ee (*R*)). The slightly lower conversion for (*R*)-**5** compared with that for (*S*)-**5** again indicates the slightly higher preference of AcCO6 to oxidize the (*S*)-enantiomer of the substrate. Notably, the isomerization of the aldehyde intermediate **8** to the more stable hydroxyacetophenone (**6**) via keto–enol tautomerization was not detected (see Figure S10). However, we observed the formation of benzylamine (**11**) as a side product (from 2% to 8%) in nearly all tests; the only exception was the reaction of *rac*-**5** at the 10 mM concentration for which quantitative conversion into **9** was detected. A possible explanation for this result is that the aldehyde intermediate **8** undergoes cleavage to benzaldehyde (**10**), which is then converted to benzylamine (**11**) by Ch1-AmDH (see Figure S10). To investigate that possibility, (*S*)-**5** (20 mM) was incubated in a HCOONH_4_ buffer (pH 8.5, 1 M, 1 mL) in the presence of AcCO6 (50 μM) and a catalase (0.1 mg mL^–1^). A negative control experiment was performed by incubating the substrate in the same reaction mixture devoid of AcCO6 and the catalase (for details, see SI section 7). The reactions in the presence of AcCO6 and the catalase led to 27% and 32% benzaldehyde (**10**) formation within 6 and 48 h of incubation, respectively. In contrast, we did not observe any formation of **10** in the negative control experiment after 48 h (see Figure S11). Therefore, benzylamine (**11**) was indeed formed by the Ch1-AmDH-catalyzed reductive amination of side product **10**.

Different enzyme loadings were tested to improve the conversions, especially in the case of (*R*)-**5** (SI section 6 and Table S8). Three sets of experiments were carried out in which the molar ratio between AcCO6 and Ch1-AmDH was varied. The reaction of (*S*)-**5** (20 mM) at a higher AcCO6 loading (70 μM) led to quantitative substrate conversion, although 11% of **11** was formed along with 89% of the desired product (*S*)-**9**. At lower AcCO6 loadings (10 or 24 μM), (*S*)-**9** was obtained with 41–77% conversion along with traces of **11** (2–4%). Therefore, the use of equimolar amounts of AcCO6 and Ch1-AmDH (50 μM each) turned out to be the optimal condition for the amination of (*S*)-**5**, as reported in the initial experiments. In contrast, the optimal conditions for the amination of (*R*)-**5** (10 mM) were found to be 70 μM AcCO6 and 35 μM Ch1-AmDH, which resulted in 98% conversion into product (*R*)-**9** and traces of **11** (2%).

In summary, we could obtain our target products (*S*)-**9** and (*R*)-**9** with high conversions (98%) and enantiomeric excesses (ee up to >99%) by tuning the substrate and enzyme loadings to enhance the formation of the desired product while also limiting the side production of benzylamine (Table 2).

**Table 2:**
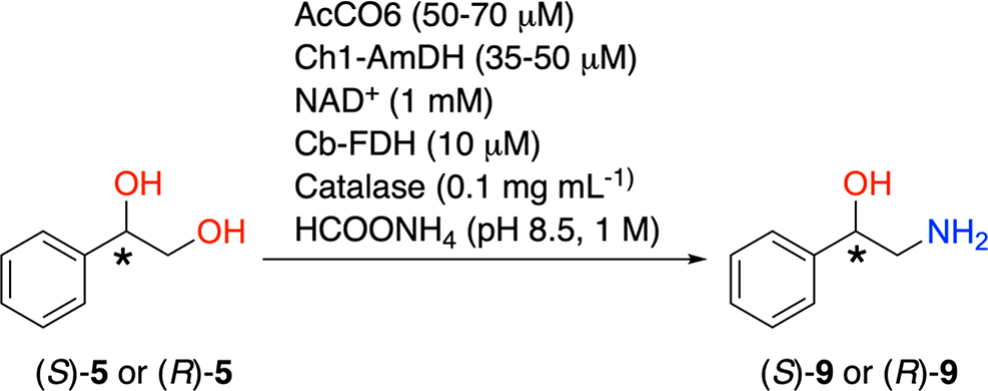
One-Pot Concurrent Oxidation–Reduction Two-Step (Disconnected) Bioamination of (*S*)- or (*R*)-**5** (10 mM) to Optically Active (*S*)- or (*R*)-**9** Catalyzed by AcCO6 Combined with Ch1-AmDH

entry	substrate	total conv. [%]^a^	conv. into **9** [%]	ee of **9** [%]^b^
1^c^	(*S*)-**5**	>99	98 ± < 1	>98 (*S*)
2^d^	(*R*)-**5**	>99	98 ± < 1	>99 (*R*)

a Reactions were performed in duplicate, and results are reported as the average of the two samples; we detected the formation of 2 ± <1% benzylamine (**11**) in the reactions with each substrate.

b Determined by RP-HPLC (C18 HD column) following the derivatization of the amino group with GITC.

c AcCO6/Ch1-AmDH 50:50 μM.

d AcCO6/Ch1-AmDH 70:35 μM.

To prove the synthetic applicability of the one-pot biocatalytic amination, the bioconversion of (*R*)-**5** to (*R*)-**9** was performed on a 104 mg scale. The product (*R*)-**9** was obtained in a 92% isolated yield with 98% purity and >99.9% ee (see the Experimental Part for details).

## 3 Conclusion

In this work, we have presented the stereoselective synthesis of both enantiomers of 2-phenylglycinol (**7**) and those of phenylethanolamine (**9**) in highly optically pure forms (>99% ee) through consecutive and divergent biocatalytic routes starting from l-phenylalanine (l-**1**) as renewable material. In the first route, l**-1** was converted into (*R*)-**5** or (*S*)-**5** at a ca. 500 mg scale with total isolated yields of 75% and 70%, respectively.

At this stage, two divergent routes were envisioned to lead to the formation of optically pure enantiomers of either 2-phenylglycinol (**7**) or phenylethanolamine (**9**). In the first route, (*R*)-**5** was converted into (*S*)-**7** or (*R*)-**7** in a 97% or 70% yield, respectively, on an analytical scale. The ca. 100 mg scale conversion of (*R*)-**5** under the same reaction conditions produced (*S*)-**7** in an 81% isolated yield with >99.4% ee. In the second route, (*R*)- or (*S*)-**5** was converted into (*R*)- or (*S*)-**9** on an analytical scale with 98% conversion. The ca. 100 mg scale conversion of (*R*)-**5** under the same reaction conditions produced (*R*)-**9** in a 92% isolated yield with >99.9% ee.

**Figure 2:**
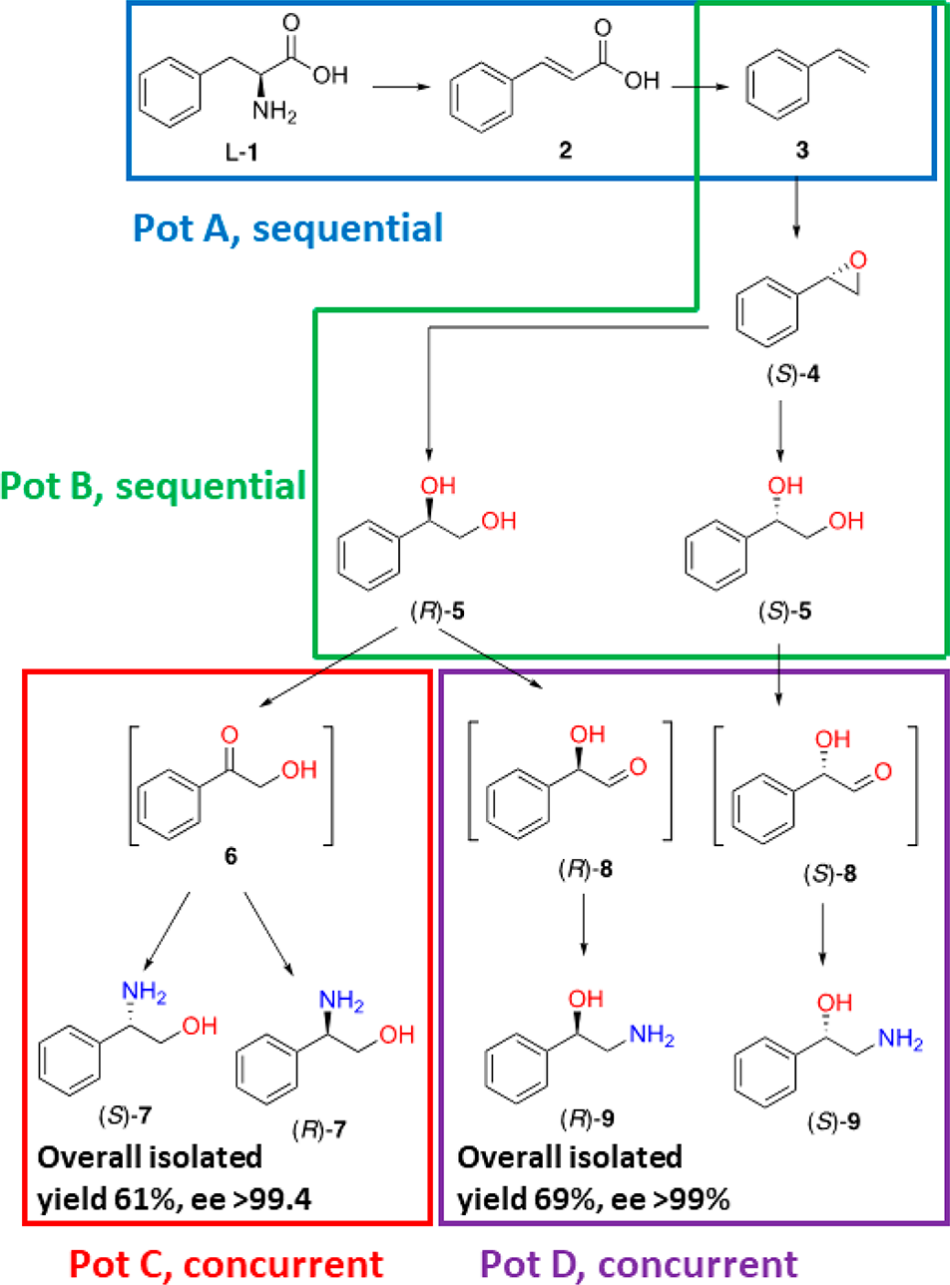
Summary of the biocatalytic pathways developed in this work and related synthetic strategies.

In summary, this work exemplifies the potential impact of linear biocatalytic cascade reactions on the highly atom efficient and sustainable syntheses of high-value chiral molecules from available and inexpensive renewable material such as l-phenylalanine. For instance, this amino acid is produced by fermentation and is a suitable starting material for further biotransformations, both in vivo and in vitro. In fact, the fermentation product mixtures of l-phenylalanine normally contain low amounts of byproducts, namely acetate, lactate, and succinate. These compounds are not known to significantly interfere with or inhibit other enzymes.^24^ However, purified l-phenylalanine can be obtained by integrating a reactive extraction step into the fermentation process, as described elsewhere at the 300 L scale.^101^ In this work, l-phenylalanine could be converted into either (*S*)-phenylglycinol or (*R*)-phenylethanolamine in a total combined yield of 61% or 69%, respectively (see Figure 2).

Notably, the process for converting l-phenylalanine into enantiomerically pure phenylethanolamine is comprised of a total of six steps performed in three pots. The reaction formally consumes only dioxygen as a simple and innocuous reagent and produces stoichiometric hydrogen carbonate as the sole byproduct, while water and ammonia molecules are formally exchanged along the process. These results also pave the way for the future metabolic engineering of *E. coli* whole-cell systems in which all the required enzymes for a certain cascade are coexpressed, thereby potentially improving the efficiency of the biochemical process.^24,102^

## 4 Experimental Part

### 4.1 Two-Pot Sequential Four-Step Cascades for the Conversion of l-Phenylalanine (l-**1**) into (*R*)-1-Phenylethane-1,2-diol ((*R*)-**5**) at a ca. 500 mg Scale

Step 1: A KPi buffer (pH 8.0, 50 mM, 150 mL) and l-**1** (20 mM, 3.1 mmol, 507 mg) were added into a 250 mL Erlenmeyer flask. Then, the pH of the mixture was adjusted to 9.0–10.0 by adding KOH (10 M), and to the mixture were added lyophilized *E. coli* whole-cells carrying overexpressed tyrosine ammonia lyase (TAL, 3 g, 20 mg mL^–1^). The reaction mixture was incubated at 30 °C and 170 rpm for 24 h. Following the removal of the cell debris by centrifugation (10 min, 14000 rpm, 18800 × *g*), an aliquot of the reaction mixture (0.5 mL) was analyzed by RP-HPLC (method A) to determine the conversion into the desired cinnamic acid intermediate (**2**). In this work, we determined the conversion using the following ratio: (observed product formation)/(observed product formation + observed remaining substrate).

Step 2: Without any intermediate workup, the pH of the reaction mixture in the same pot from step 1 was lowered to 6.5–7.0 by adding concentrated HCl. Next, to the mixture were added lyophilized *E. coli* cells carrying overexpressed ferulic acid decarboxylase (FDC1/tPAD1, 3 g, 20 mg mL^–1^). The reaction mixture was incubated at 30 °C and 170 rpm for 24 h. Following removal of the cell debris by centrifugation (10 min, 14000 rpm, 18800 × *g*), an aliquot of the reaction mixture (0.5 mL) was analyzed by RP-HPLC (method A) to determine the conversion into the styrene (**3**) . Next, **3** was extracted with *n*-heptane (3 × 50 mL), and the obtained organic solution was used directly in the subsequent step.

Step 3: A KPi buffer (pH 8.0, 50 mM, 150 mL), lyophilized *E. coli* cells carrying coexpressed chimeric styrene monooxygenase and formate dehydrogenase (Fus-SMO/FDH, 5 mg mL^–1^), NAD^+^ (1 mM), FAD (50 μM), HCOONa (100 mM, 5 equiv), and a catalase (0.1 mg mL^–1^) were added in a 500 mL tribaffled flask. Then, to the mixture was added the solution of **3** in *n*-heptane (150 mL) obtained from step 2. The reaction mixture was incubated at 30 °C and 200 rpm for 20 h. The conversion was monitored by GC-FID (method A) using an aliquot of the reaction mixture. When the conversion was quantitative, the reaction proceeded to step 4.

Step 4: Lyophilized *E. coli* cells carrying overexpressed epoxide hydrolase (St(R)-EH, 3 g, 20 mg mL^–1^) were added to the same pot from step 3 without any intermediate workup. The reaction mixture was further incubated at 30 °C and 170 rpm for 24 h. The *n*-heptane phase was then separated from the aqueous phase. The latter phase was saturated with solid NaCl and extracted with MTBE (3 × 100 mL). The combined organic phase was dried over anhydrous MgSO_4_ and concentrated under reduced pressure to yield product (*R*)-**5** (320 mg, 75% total isolated yield calculated from the l-phenylalanine (l-**1**) starting material; ee > 99% (*R*); 93% purity). The conversion and purity of the isolated product were analyzed by GC-FID (method A), and the enantiomeric excess was determined using chiral NP-HPLC (method B); see SI section 3.3 and Figures S4 and S5. ^1^H NMR (see Figure S6) spectra were recorded after column chromatography with petroleum ether and ethyl acetate (1:1, v v^–1^) as the eluent (*R*_f_ = 0.3), which afforded the quantitative yield of purification.

### 4.2 One-Pot Simultaneous Interconnected Two-Step Cascade for the Conversion of (*R*)-1-Phenylethane-1,2-diol ((*R*)-**5**) into (*S*)-2-Phenylglycinol ((*S*)-**7**) at a ca. 100 mg Scale

An ammonium formate buffer (2 M, pH 8.5, 37.5 mL), H_2_O (28 mL), NAD^+^ (1 mM, 49 mg), PLP (1 mM, 19 mg), d-alanine (50 mM, 322 mg), and substrate (*R*)-**5** (10 mM, 101 mg) were added to a 250 mL Erlenmeyer flask. The pH was adjusted to 8.5 with ammonia. Then, to the mixture were added Bs-AlaDH (20 μM), Aa-ADH (70 μM), and At-ωTA (35 μM). The total reaction volume was 73 mL. The reaction mixture was incubated at 30 °C for 70 h. The aqueous reaction mixture was basified with KOH (10 M, 9 mL), saturated with solid NaCl, and extracted with EtOAc (2 × 40 mL). Following the drying of the combined organic phases over anhydrous MgSO_4_, the organic phase was concentrated under reduced pressure to yield product (*S*)-**7** (orange oil, 81% isolated yield (81 mg), 98% purity, ee > 99.4%). The conversion and purity of the isolated product were measured by GC-FID (method A), ^1^H NMR, and ^13^C NMR (see SI section 8 and Figures S12, S15, and S16). The enantiomeric excess was determined by RP-HPLC following derivatization with GITC (method D); see Figure S13.

### 4.3 One-Pot Simultaneous Disconnected Two-Step Cascade for the Conversion of (*R*)-1-Phenylethane-1,2-diol ((*R*)-**5**) into (*R*)-Phenylethanolamine ((*R*)-**9**) at a ca. 100 mg Scale

An ammonium formate buffer (2 M, pH 8.5, 37.5 mL), H_2_O (16 mL), NAD^+^ (1 mM, 49 mg), a catalase (0.1 mg mL^–1^, 7.3 mg), and substrate (*R*)-**5** (10 mM, 104 mg) were added to a 250 mL Erlenmeyer flask. The pH was adjusted to 8.5 with ammonia. Then, to the mixture were added Cb-FDH (10 μM), AcCO6 (70 μM), and Ch1-AmDH (35 μM). The total reaction volume was 73 mL. The reaction mixture was incubated at 30 °C for 70 h. The aqueous reaction mixture was basified with KOH (10 M, 9 mL), saturated with solid NaCl, and extracted with EtOAc (2 × 40 mL). Following the drying of the combined organic phases over anhydrous MgSO_4_, the organic phase was concentrated under reduced pressure to yield product (*R*)-**9** (yellow oil, 92% isolated yield (95 mg), 98% purity, ee > 99.9%). The conversion and purity of the isolated product were measured by GC-FID (method A), ^1^H NMR, ^13^C NMR (see SI section 8 and Figures S12, S17, and 18). The enantiomeric excess was determined by RP-HPLC following derivatization with GITC (method D); see Figure S14.

## Supporting Information Available

Details on the enzymes used in this work; general procedures and references for enzyme expression and purification; experimental procedures for the cascade reactions and other experiments on an analytical scale; analytical procedures for the determination of conversions, enantiomeric excesses, and the derivatization of compounds; HPLC and GC chromatograms; and NMR spectra (PDF)
