# Supplementary material for: High-Yield Synthesis of Enantiopure 1,2-Amino Alcohols from l-Phenylalanine via Linear and Divergent Enzymatic Cascades
Source: Org Process Res Dev. 2022 Mar 28;26(7):2085–95. doi: 10.1021/acs.oprd.1c00490 (PMC9295148; doi:10.1021/acs.oprd.1c00490)
Supplement: Supplementary file 1 — op1c00490_si_001.pdf [file op1c00490_si_001.pdf]

Supporting Information for:

High yield synthesis of enantiopure 1,2-amino alcohols from l-phenylalanine via linear and divergent enzymatic cascades

Maria L. Corrado,[a] Tanja Knaus,*,[a] Ulrich Schwaneberg[b] and Francesco G. Mutti*,[a]

[a] Van 't Hoff Institute for Molecular Sciences, University of Amsterdam, HIMS-Biocat, Science Park 904, 1098 XH Amsterdam, the Netherlands. E-mail: f.mutti@uva.nl; t.knaus@uva.nl;
[b] Institute of Biotechnology, RWTH Aachen University, Worringerweg 3, 52074, Aachen, Germany.

Table of Contents

1. Enzymes used in this study 3

1.1 General procedure for enzymes expression and purification 4

2. One-pot sequential two-step conversion of 1 into 3 5

3. One-pot sequential two-step conversion of 3 into (*R*)-5 or (*S*)-5 6

3.1 Initial study for the conversion of 3 into either (*R*)-5 or (*S*)-5 catalyzed by *E. coli*/Fus-SMO/FDH and *E. coli*/EHs in different organic solvents and different ratio with aqueous buffer 6

3.2 Initial study for the preparative scale enzymatic synthesis of chiral diols 5 starting from 3 7

3.3 Optimized preparative scale enzymatic synthesis of chiral diols 5 implemented in the two-pot sequential four-step cascades starting from 1 7

4. Screening of secondary $NAD(P)^+$-dependent ADHs for the biocatalytic oxidation of either *rac*-5 or (*S*)-5 or (*R*)-5 into 6 catalyzed by $NAD(P)^+$-dependent ADHs in Tris-HCl buffer 9

5. One-pot concurrent two-step cascade for the conversion of either *rac*-5 or (*S*)-5 or (*R*)-5 into either (*S*)-7 or (*R*)-7 catalyzed by ADHs combined with various stereocomplementary ωTAs in $HCOONH_4$ buffer 11

6. One-pot concurrent disconnected two-step cascade for the conversion of either *rac*-5 or (*S*)-5 or (*R*)-5 into either (*S*)-9 or (*R*)-9 catalyzed by AcCO6 combined with Ch1-AmDH 12

7. Study on the formation of benzylamine (11) as side-product during the oxidation of 5 14

8. Biocatalytic conversions of (*R*)-1-phenylethane-1,2-diol ((*R*)-5) into either (*S*)-2-phenylglycinol ((*S*)-7) or (*R*)-phenylethanolamine ((*R*)-9) at ca. 100 mg scale 16

9. Representative GC-FID and HPLC chromatograms for the biocatalytic aminations of diols 5 21

10. General derivatization procedure for the determination of the enantiomeric excess by RP-HPLC 27

11. Analytical methods 27

12. References 28

List of abbreviations

TAL	Tyrosine ammonia lyase
FDC1/tPAD1	Ferulic acid decarboxylase
ADH	Alcohol dehydrogenase
AlaDH	Alanine dehydrogenase
BDHA	2,3-butanediol dehydrogenase
AOx	alcohol oxidase
CIP	Cahn-Ingold-Prelog
EDTA	Ethylenediaminetetraacetic acid
EH	Epoxide hydrolase
ee	Enantiomeric excess
FDH	Formate dehydrogenase
Fus-SMO	Chimeric styrene monooxygenase (i.e., fused StyA and StyB enzymes)
GITC	2,3,4,6-Tetra-O-acetyl-β-D-glucopyranosyl isothiocyanate
$HCOONH_4$	Ammonium formate
KH_2PO_4	Monopotassium phosphate
KPi	Potassium phosphate buffer salts
LB	Luria-Bertani Broth
MTBE	*tert*-Butyl methyl ether
n.c.	No conversion
n.d.	Not detected
n.m.	Not measured
NaCl	Sodium chloride
$NAD(P)^+$	Nicotinamide adenine dinucleotide (phosphate)
NP-HPLC	Normal-phase HPLC
PLP	pyridoxal 5'-phosphate
RP-HPLC	Reverse-phase HPLC
SDS-PAGE	Polyacrylamide gel electrophoresis
ωTA	ω-Transaminase
Tris-HCl	Tris(hydroxymethyl)aminomethane buffer salts

General information: Alcohol dehydrogenases (ADHs: Aa, Lbv, Pp, Sy, BDHA, Ls, Te-v1, Te-v2, Te-v3, Lb and Rs), amine dehydrogenases (AmDH: Ch1-AmDH and Rs-PhAmDH), fused styrene monooxygenase (Fus-SMO) and epoxide hydrolases (EHs: Sp(*S*)-EH and St(*R*)-EH) were expressed and, when stated, purified according to the procedures described in literature.[1] The ωTAs used in this study were expressed and purified as reported in literature.[2] Ferulic acid decarboxylase (FDC1/tPAD1) was expressed as reported in literature.[3] AcCO6 was expressed and purified according to the procedures reported in literature.[4] Rs-TAL was expressed according to the procedure reported in literature.[5-6] More details about the used recombinant enzymes and the strains of origin are reported in Table S1. General procedures for the recombinant enzyme expression and, when stated, purification are also reported in section 1.1. Compounds *rac*-**5** and **6** were purchased by TCI. Reference compounds **7** and **9** were purchased by Fluorochem.

1. Enzymes used in this study

Table S1. List of enzymes employed in this study

Name	Source/Comment	Used form	Ref
Fus-SMO	Fused SMO from *Pseudomonas* sp. VLB120	lyophilized whole cells	7
Fus-SMO (1) + Cb-FDH (2)	Fused SMO from *Pseudomonas* sp. VLB120 coexpressed with FDH from *Candida boidinii*	lyophilized whole cells	7
Sp(S)-EH	EH from *Sphingomonas* sp. HXN200	lyophilized whole cells	8
St(R)-EH	EH from *Solanum tuberosum*	lyophilized whole cells	8
Rs-ADH	ADH from *Ralstonia* sp.	lyophilized whole cells	9
Aa-ADH	ADH from *Aromatoleum aromaticum*	purified	10-11
Pp-ADH	ADH from *Paracoccus pantotrophus* DSM 11072	lyophilized whole cells	12
Sy-ADH	ADH from *Shingobium yanoikuyae* DSM 6900	lyophilized whole cells	13
Te-ADH-v1	ADH I86A variant from *Thermoanaerobacter ethanolicus*	lyophilized whole cells	14
Te-ADH-v2	ADH W110A variant from *Thermoanaerobacter ethanolicus*	lyophilized whole cells	14
Te-ADH-v3	ADH I86A W110A variant from *Thermoanaerobacter ethanolicus*	lyophilized whole cells	14
Lb-ADH	ADH from *Lactobacillus brevis*	purified	15
Lbv-ADH	ADH variant from *Lactobacillus brevis*	purified	16-17
Ls-ADH	ADH from *Leifsonia* sp.	lyophilized whole cells or purified	18
Bs-BDHA	ADH from *Bacillus subtilis* BGSC1A1	purified	19-20
Ch1-AmDH	AmDH from Chimeric AmDH	purified	17, 21
Rs-PhAmDH	AmDH variant from *Rhodoccoccus sp.*	purified	22-23
LE-AmDH	AmDH variant from Ɛ-(deaminating) lysine dehydrogenase	purified	24
Cb-*FDH*	FDH from *Candida boidinii*	purified	23
YcnD	NAD(P)H-dependent oxidase from *Bacillus subtilis*	purified	14, 25
NOx	NADH-dependent oxidase from *Streptococcus mutans*	purified	26-27
Bs-AlaDH	AlaDH from *Bacillus sphaericus*	purified	28
At(R)-ωTA	ωTA from *Aspergillus terreus*	purified	29-30
Cv(S)- ωTA	ωTA from *Chromobacterium violaceum* DSM 30191	purified	31
Bm(S)-ωTA	ωTA from *Bacillus megaterium* SC6394	purified	32-33
Vf(S)-ωTA	ωTA from *Vibrio fluvialis*	lyophilized whole cells	34-35
Ac(S)-ωTA	ωTA from *Arthrobacter citreus*	purified	33
AcCO6	AOx variant from choline oxidase *Arthrobacter chlorophenolicus*	purified	4
Rs-TAL	TAL from *Rhodobacter sphaeroides*	Lyophilized whole cells	5-6
FDC1-tPAD1	FDC1/tPAD1 from *Saccharomyces cerevisiae*	Lyophilized whole cells	3

1.1 General procedure for enzymes expression and purification

Expression of the enzymes: For recombinant expression, 800 mL of LB medium supplemented with the appropriate antibiotic (100 µg mL^{-1} ampicillin or 50 µg mL^{-1} kanamycin) were inoculated with 15 mL of an overnight culture harboring the desired vector with genes for the expression of the enzyme. *E. coli* BL21 DE3 cells were used as expression host organism in this study. Cells were grown at 37 °C until an OD600 of 0.6 to 1 was reached and expression of protein was induced by the addition of IPTG. Protein expression was carried out overnight and after harvesting of the cells (4 °C, 4500 rpm, 10 min), the remaining cell pellets were washed with buffer. For the preparation of lyophilized *E. coli* whole cells, we used: 50 mM Tris-HCl buffer at pH 8.0 for ADHs and 50 mM KPi at pH 8.0 for ωTA, EHs, TAL and FDC1-tPAD1. For the preparation of the cell lysate for further enzyme purification by affinity chromatography, we used lysis buffer as reported in the next paragraph.

Purification by Nickel affinity chromatography: His_6-tagged proteins were resuspended in lysis buffer (50 mM KH_2PO_4, 300 mM NaCl, 10 mM imidazole, pH 8.0) prior to cell disruption. Protein purification was performed by Ni-NTA affinity chromatography using pre-packed Ni-NTA HisTrap HP columns (GE Healthcare), previously equilibrated with lysis buffer. After loading of the filtered lysate, the column was washed with sufficient amounts of wash buffer (50 mM KH_2PO_4, 300 mM NaCl, 25 mM imidazole, pH 8.0), and bound protein was recovered with elution buffer (50 mM KH_2PO_4, 300 mM NaCl, 200 mM imidazole, pH 8.0). Enzymes purity was analyzed by SDS-PAGE and fractions showing >95% purity were combined and dialyzed overnight against Tris-HCl buffer (6 L, pH 8.0, 20 mM). The enzyme solutions were concentrated and their concentration was determined spectrophotometrically based on their extinction coefficient at 280 nm. In the case of transaminases, protein concentration was determined according to Bradford assay using ovalbumin as standard protein.[2]

Purification by Strep affinity chromatography: 3N-strep-tagged proteins were resuspended in binding buffer (100 mM Tris-HCl, 150 mM NaCl and 1 mM EDTA, pH 8.0) prior to cell disruption and protein purification was performed by Strep affinity chromatography using pre-packed columns with StrepTactin Sepharose (GE Healthcare), previously equilibrated with binding buffer. The supernatant was split in four aliquots after filtration (typically 4 x 10 mL). Each aliquot was purified in a separated step. After loading of the aliquot and washing with buffer, 3N-strep-tagged bound protein was recovered with elution buffer (100 mM Tris-HCl, 150 mM NaCl, 1 mM EDTA and 2.5 mM desthiobiotin, pH 8.0). Purity was analyzed by SDS-PAGE and fractions showing >95% purity were combined and dialyzed overnight against Tris-HCl buffer (6 L, pH 8.0, 20 mM). The enzyme solutions were concentrated and their concentration was determined spectrophotometrically based on their extinction coefficient at 280 nm.

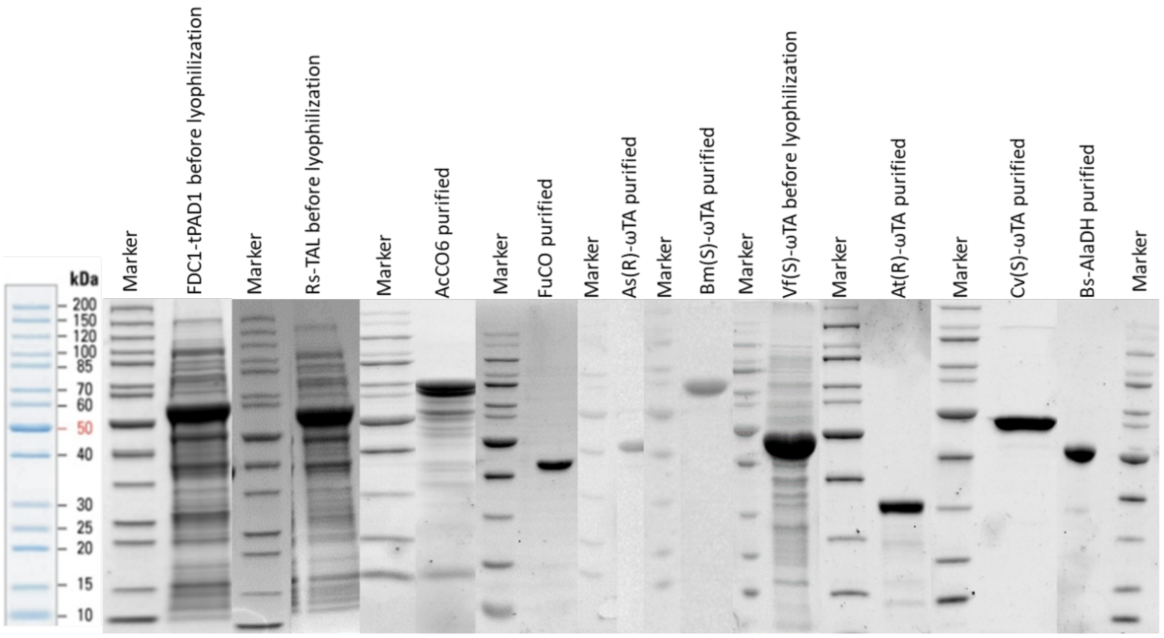

Figure S1. SDS-PAGE of lyophilized whole cells (pellet before lyophilization) or purified enzymes. Marker: PageRuler™ Unstained Protein Ladder (ThermoFisher Scientific).

2. One-pot sequential two-step conversion of 1 into 3

The experimental procedures are reported in the main manuscript.

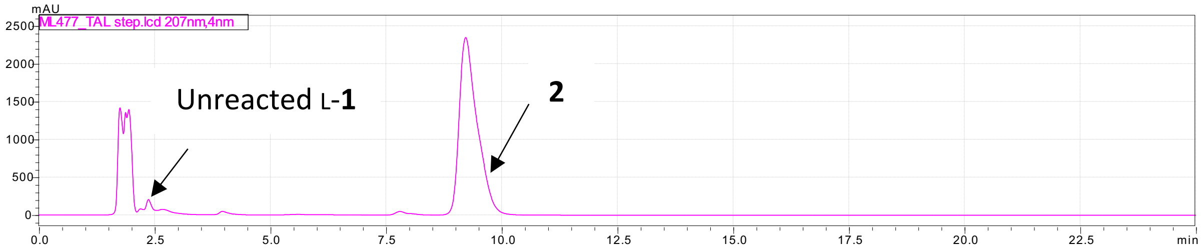

Figure S2. Representative RP-HPLC analysis for the conversion of L-phenylalanine (L-**1**) to cinnamic acid intermediate (**2**) catalyzed by lyophilized *E. coli* whole cells carrying overexpressed Rs-TAL. Note: peak at 1.5–2 min attributed to buffer salt/*E. coli* whole cells.

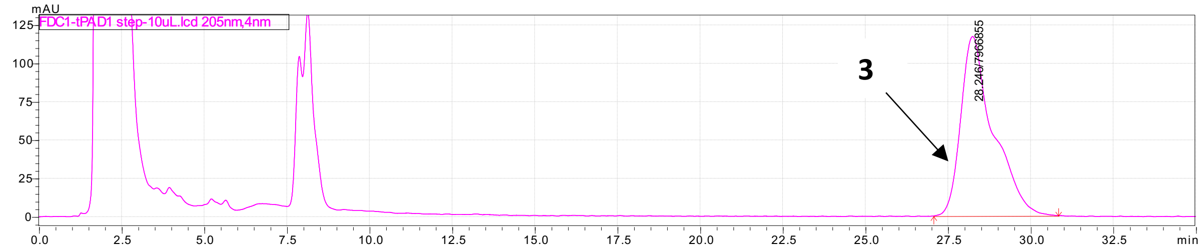

Figure S3. Representative RP-HPLC analysis for the conversion of cinnamic acid (**2**) into styrene (**3**) catalyzed by lyophilized *E. coli* whole cells carrying overexpressed FDC1-tPAD1. Note: peaks at 2.5 min and 8 min are attributed to buffer salt/*E. coli* whole cells.

3. One-pot sequential two-step conversion of 3 into (*R*)-5 or (*S*)-5

3.1 Initial study for the conversion of 3 into either (*R*)-5 or (*S*)-5 catalyzed by *E. coli*/Fus-SMO/FDH and *E. coli*/EHs in different organic solvents and different ratio with aqueous buffer

In this initial study, lyophilized *E. coli* whole cells carrying overexpressed Fus-SMO (20 mg mL^{-1}) and lyophilized *E. coli* whole cells carrying overexpressed EHs (20 mg mL^{-1}) were added together at the start of the cascade. Cells were rehydrated in KPi buffer (pH 8.0, 50 mM; 0.5–1 mL according to Table S2) in a glass vial (4 mL). The buffer already contained NAD^+ (1 mM), HCOONa (100 mM) and FAD (50 µM). Then Cb-FDH (10 µM) was added followed by substrate **3** (20 mM, otherwise stated). A second phase, consisting of an organic solvent (2-50%), was also added in selected experiment (see Table S2). The mixture was shaken on an orbital shaker at 30 °C, 180 rpm for 24 h. The aqueous layer was saturated with solid NaCl and extracted with MTBE (2 x 500). The combined organic phase (MTBE and reaction solvent, when the latter was used) was dried over $MgSO_4$ and analyzed by GC-FID and chiral NP-HPLC.

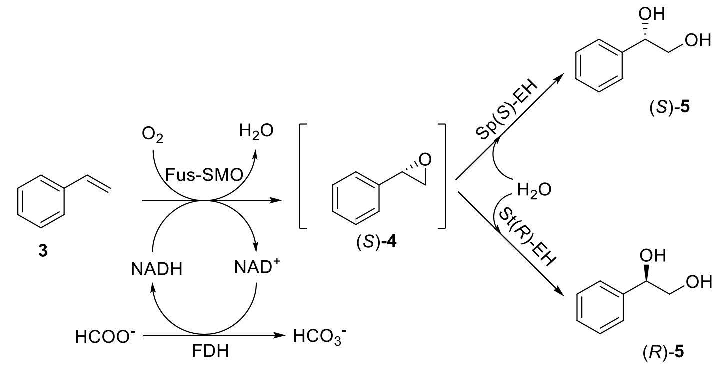

Table S2. Bioconversion of **3** (20 mM) to chiral **5** catalyzed by Fus-SMO coupled with one of two stereocomplementary EHs in different solvent systems.

Entry	EH	KPi buffer (pH 8.0, 50 mM)	Organic solvent	Conversion [%]	*ee* [%][a]
1	Sp(*S*)-EH	0.5 mL	0.5 mL Hexane	>99	>99 (*S*)
2	Sp(*S*)-EH	1 mL	-	>79	99 (*S*)
3	Sp(*S*)-EH	950 µL	50 µL styrene (5%)	2.5	n.m.
4	Sp(*S*)-EH	0.5 mL	0.5 mL heptane	>90	98 (*S*)
5	St(*R*) EH	0.5 mL	0.5 mL hexane	>99	97 (*R*)
6	St(*R*)-EH	1 mL	-	>88	97 (*R*)
7	St(*R*)-EH	950 µL	50 µL styrene (5%)	3	n.m.
8	St(*R*)-EH	0.5 mL	0.5 mL heptane	>99	97 (*R*)
9	Sp(*S)*-EH	980 µL	20µL decane (2%)	>80	99 (*S*)
10	Sp(*S*)-EH	960 µL	40µL decane (4%)	>72	>98 (*S*)
11	Sp(*S*)-EH	940 µL	60µL decane (6%)	>78	99 (*S*)
12	Sp(*S*)-EH	920 µL	80µL decane (8%)	>63	99 (*S*)
13	Sp(*S*)-EH	900 µL	100µL decane (10%)	>59	99 (*S*)

14	St(*R*)-EH	980 µL	20µL decane (2%)	>66	97 (*R*)
15	St(*R*)-EH	960 µL	40µL decane (4%)	>60	97 (*R*)
16	St(*R*)-EH	940 µL	60µL decane (6%)	>55	97 (*R*)
17	St(*R*)-EH	920 µL	80µL decane (8%)	>54	98 (*R*)
18	St(*R*)-EH	900 µL	100µL decane (10%)	>70	97 (*R*)

Reactions were all performed in duplicate and the results reported are the average of the two samples; [a] analysed by chiral HPLC (OD-H column); n.m. = not measured

3.2 Initial study for the preparative scale enzymatic synthesis of chiral diols 5 starting from 3

General remark: The concentrations of coenzymes and co-substrate are always calculated on the volume of the aqueous phase, whereas the concentration of the substrate is referred to the volume of the organic phase. Furthermore, the preparative scale reactions were performed under the optimized conditions as we have previously reported for similar substrates.[1]

Reaction conditions: Lyophilized *E. coli* cells co-expressing Fus-SMO and Cb-FDH (250 mg, 5 mg mL^{-1}) were rehydrated in KPi buffer (50 mL, 50 mM, pH 8.0) in a baffled Erlenmeyer flask (500 mL). After that, NAD^+ (1 mM), HCOONa (5 eq.), FAD (50 µM) and catalase (0.1 mg mL^{-1}) were added. *n*-Heptane (50 mL; 1:1 volumetric ratio with the buffer) was used as organic solvent in the biphasic reaction medium. Then, the biocatalytic reactions were initiated by the addition of substrate **3** (50 mM, 2.5 mmol). The reactions were incubated at 30 °C and 200 rpm on an orbital shaker. After 6 hours, lyophilized *E. coli* cells expressing either Sp(S)-EH or St(R)-EH (1 g, 20 mg mL^{-1}) were added and the reactions were further incubated at 30 °C and 170 rpm on an orbital shaker for 30 h. *n*-Heptane was separated from the aqueous phase. The latter phase was saturated with solid NaCl and the organic compounds extracted with MTBE (3 x 25 mL). The combined MTBE layers were dried over $MgSO_4$ and the organic solvent was removed under reduced pressure yielding the desired diols in ca. 67% isolated yield (240 mg; *ee* >99 *R*; *ee* 98–99% *S*). Conversions and the purity of the isolated products were determined by GC-FID, while the enantiomeric excess was analyzed by NP-HPLC.

3.3 Optimized preparative scale enzymatic synthesis of chiral diols 5 implemented in the two-pot sequential four-step cascades starting from 1

Experimental procedure is reported in main manuscript, experimental part. Chromatograms are reported below.

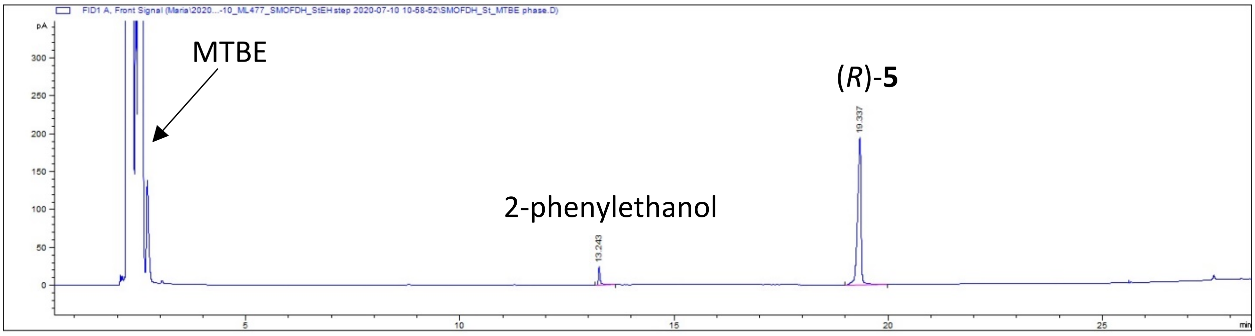

Figure S4. GC-FID analysis for the conversion of styrene **3** into (*R*)-1-phenylethane-1,2-diol ((*R*)-**5**) catalyzed by the lyophilized *E. coli* whole cells co-expressing Fus-SMO and FDH, and lyophilized *E. coli* whole cells expressing St(*R*)-EH.

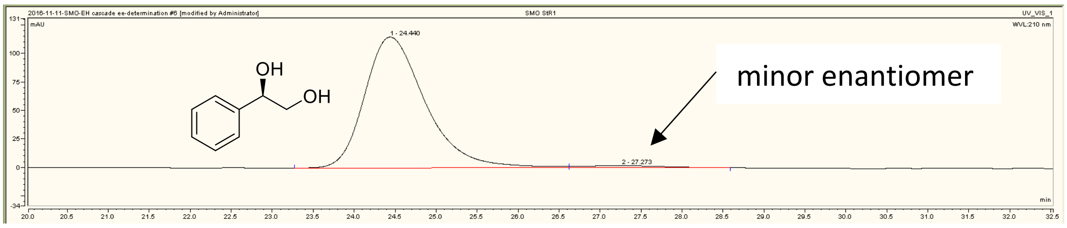

Figure S5. Chiral NP-HPLC analysis of the obtained (*R*)-**5**.

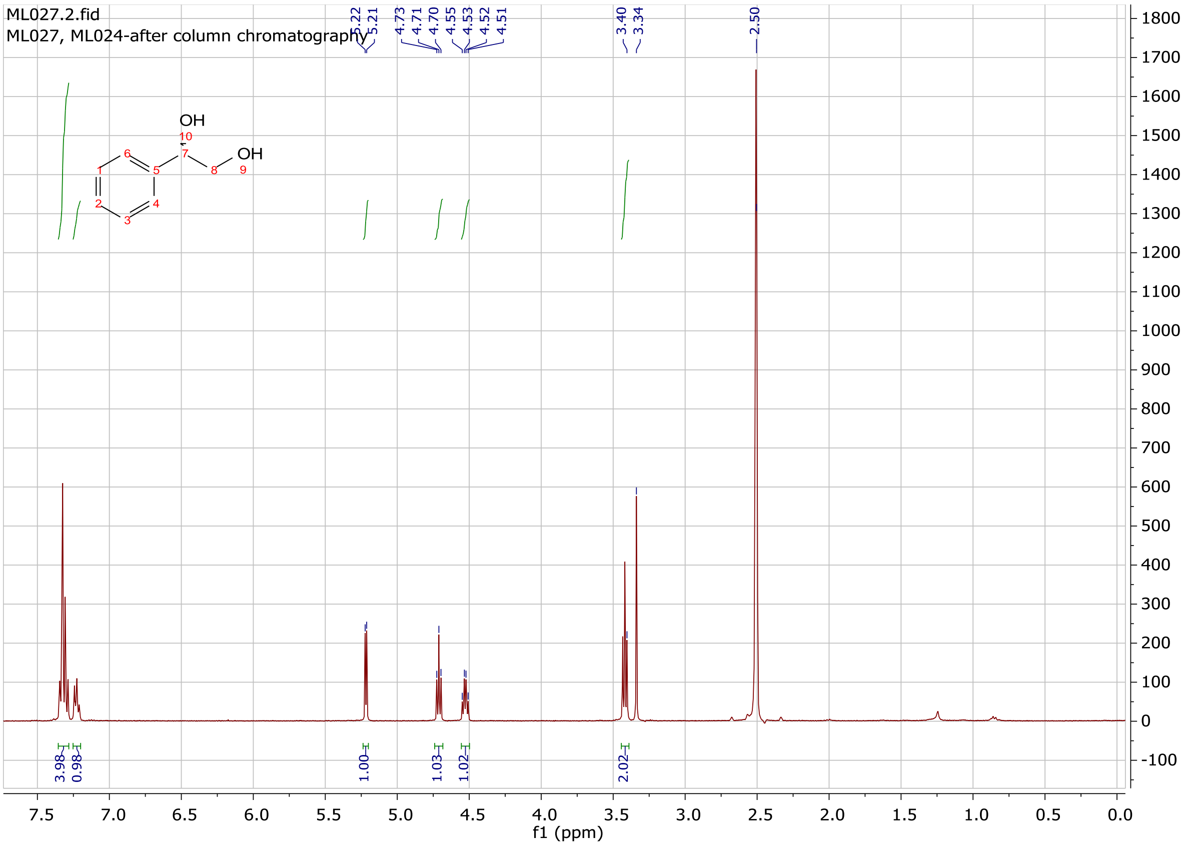

Figure S6. ^{1}H-NMR of the obtained (*R*)-**5**.

4. Screening of secondary NAD(P)$^+$-dependent ADHs for the biocatalytic oxidation of either *rac*-5 or (*S*)-5 or (*R*)-5 into 6 catalyzed by NAD(P)$^+$-dependent ADHs in Tris-HCl buffer

Reaction conditions using lyophilized *E. coli* whole cells expressing one of the following NAD(P)$^+$-dependent ADHs: Sy-ADH, Pp-ADH, Bs-BDHA, Te-ADH-v1, Te-ADH-v2, Te-ADH-v3 and Rs-ADH. Lyophilized *E. coli* cells (20 mg mL^{-1}) were rehydrated in an Eppendorf tube (2 mL) in Tris-HCl buffer (1 mL, pH 7.5, 50 mM) containing NAD(P)$^+$ (1 mM). NOx (10 µM)—for NAD$^+$-dependent ADHs—or YcnD (10 µM)—for NADP$^+$-dependent ADHs—was also added for cofactor regeneration. Substrate *rac*- or (*S*)- or (*R*)-**5** (10-20 mM) was added as last. The mixtures were incubated at 30 °C, 170 rpm for 24 h on an orbital shaker and, after saturation of the aqueous layer with solid NaCl, the organic compounds were extracted with EtOAc (2 x 500 µL). The organic layers were dried over $MgSO_4$ and analyzed by GC-FID.

Reaction conditions using lyophilized *E. coli* whole cells expressing the NAD$^+$-dependent Ls-ADH. Lyophilized *E. coli* cells (10 mg mL^{-1}) were rehydrated in an Eppendorf tube (2 mL) in KPi buffer (1 mL, pH 6.5, 100 mM) containing NAD$^+$ (1 mM). NOx (10 µM) was also used for cofactor regeneration and *rac*- or (*S*)- or (*R*)-**5** (20 mM) was added as last. The mixture was incubated at 40 °C, 170 rpm for 24 h on an orbital shaker. After saturation of the aqueous layer with solid NaCl, extraction was performed with MTBE (2 x 500 µL). The organic layer was dried over $MgSO_4$ and analyzed by GC-FID.

Reaction conditions using purified ADHs: Lbv-ADH, Aa-ADH and Lb-ADH.

General information: in selected cases Aa-ADH and Lbv-ADH were combined in the same pot. In these cases, both enzymes were used in 25 µM concentration each.

Tris-HCl buffer (1 mL, pH 7.5, 50 mM) was added to an Eppendorf tube (2 mL) containing either NAD$^+$ or NADP$^+$ (1 mM, 0.1 eq.); NOx (10 µM)—for NAD$^+$-dependent ADHs—or YncD (10 µM)—for NADP$^+$-dependent ADHs—were also added. As last, the tested ADH (50 µM) was added followed by substrate *rac*- or (*S*)- or (*R*)-**5** (10-20 mM). The mixture was incubated at 30 °C, 170 rpm for 24 h on an orbital shaker. After saturation of the aqueous layer with solid NaCl, extraction was performed with EtOAc (2 x 500 µL). The organic layer was dried over $MgSO_4$ and analyzed by GC-FID.

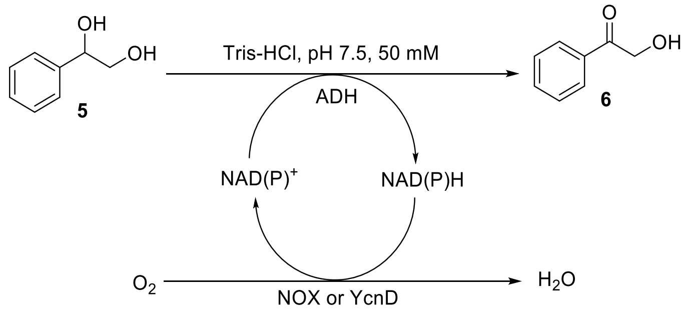

Figure S7. General scheme for the biocatalytic oxidation of **5** to **6**.

Table S3. Screening of secondary NAD(P)$^+$-dependent ADHs for the biocatalytic oxidation of *rac*-**5** (20 mM) into **6** in Tris-HCl buffer (pH 7.5, 50 mM)

Entry	ADH	6 [%]	Coenzyme	Catalyst form[c]
1	Aa-ADH	71	NAD^+	Purified
2	Pp-ADH [b]	n.c.	NAD^+	Lyophilized whole *E.coli* cells
3	Sy-ADH [b]	n.c.	NAD^+	Lyophilized whole *E.coli* cells
4	Lbv-ADH	81	NAD^+	Purified
5	Bs-BDHA	45	NAD^+	Lyophilized whole *E.coli* cells
6	Ls-ADH[b]	14±1	NAD^+	Lyophilized whole *E.coli* cells
7	Aa-ADH[a] +Lbv-ADH[a]	89	NAD^+	Purified
8	Te-ADH_{v1}	n.c.	$NADP^+$	Lyophilized whole *E.coli* cells
9	Te-ADH_{v2}	n.c.	$NADP^+$	Lyophilized whole *E.coli* cells
10	Te-ADH_{v3}	n.c.	$NADP^+$	Lyophilized whole *E.coli* cells
11	Lb-ADH	58	$NADP^+$	Purified
12	Rs-ADH	24	$NADP^+$	Lyophilized whole *E.coli* cells

[a] Used in 25 µM; [b] Reactions were performed in duplicate and the reported conversion is the average of the two samples; n.c. = no conversion; [c] for details see section 1.

Table S4. Screening of secondary NAD^+-dependent ADHs for the biocatalytic oxidation of either (*S*)-**5** (10 mM) or (*R*)-**5** (10 mM) into **6** in Tris-HCl buffer (pH 7.5, 50 mM)

Entry	Substrate	ADH	Coenzyme	6 [%]	Catalyst form[b]
1	(*S*)-**5**	Aa-ADH[a]	NAD^+	n.c.	Purified
2	(*S*)-**5**	Lbv-ADH	NAD^+	>99	Purified
3	(*S*)-**5**	Bs-BDHA[a]	NAD^+	n.c.	Lyophilized whole *E.coli* cells
4	(*R*)-**5**	Aa-ADH [a]	NAD^+	84±8	Purified
5	(*R*)-**5**	Lbv-ADH [a]	NAD^+	1±<1	Purified
6	(*R*)-**5**	Bs-BDHA	NAD^+	69	Lyophilized whole *E. coli* cells

[a]Reactions were performed in duplicate and the reported conversion is the average of the two independent samples; n.c. = no conversion; [b] for details see section 1.

5. One-pot concurrent two-step cascade for the conversion of either *rac*-5 or (*S*)-5 or (*R*)-5 into either (*S*)-7 or (*R*)-7 catalyzed by ADHs combined with various stereocomplementary ωTAs in $HCOONH_4$ buffer

General information: in selected cases Aa-ADH and Lbv-ADH were combined in the same pot. In these cases, each enzyme was used in 25 µM.

$HCOONH_4$ buffer (pH 8.5, 1 M; 0.5 mL) was added to an Eppendorf tube (1.5 mL) and supplemented with NAD^+ (1 mM), PLP (1 mM), D- or L-Alanine (50 mM, 5 eq.) and Bs-AlaDH (20 µM). Then, ADH (50 µM otherwise stated in Table S5-6) and ωTA (50 µ otherwise stated in Table S5-6) were added followed by substrate **5** (10 mM). The mixture was incubated at 30 °C, 170 rpm for 48 h on an orbital shaker and, after that, quenched with 10 M KOH (100 µL). The aqueous layer was saturated with solid NaCl and the organic compounds extracted with EtOAc (1 x 500 µL). The organic layer was dried over $MgSO_4$ and analyzed by GC-FID to determine the conversion, while the enantiomeric excesses was analyzed by RP-HPLC after derivatization with a chiral reagent (GITC).

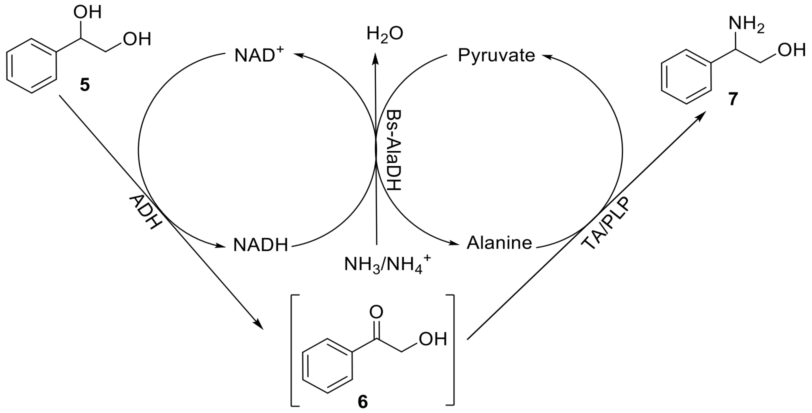

Figure S8. General scheme for the biocatalytic amination of **5** to **7**.

Table S5. Biocatalytic oxidation/transamination of chiral (*S*)- or (*R*)-**5** (10 mM) catalyzed by ADHs (50 µM) coupled with one of two stereocomplementary ωTAs (50 µM) in $HCOONH_4$ (pH 8.5, 1 M, 30 °C).

Entry	Substrate	ADH	ωTA	Conv.[%]	7 [%]	*ee* 7 [%][a]	6 [%]
1	(*S*)-**5**	Lbv-ADH	Cv-ωTA	3±<1	3±<1	n.m.	n.d.
2	(*S*)-**5**	Lbv-ADH	At-ωTA	21±1	21±1	>99 (*S*)[b]	n.d.
3	(*R*)-**5**	Aa-ADH	Cv-ωTA	11±3	11±3	82.5 (*R*)[b]	n.d.
4	(*R*)-**5**	Aa-ADH	At-ωTA	93±1	93±1	>99 (*S*)[b]	n.d.
5	(*R*)-**5**	Bs-BDHA	Cv-ωTA	n.c.	n.d.	n.m.	n.d.
6	(*R*)-**5**	Bs-BDHA	At-ωTA	92±<1	91±<1	>99 (*S*)[b]	1±<1

[a] analyzed by RP-HPLC (C18 HD column) after derivatization of the amino group with GITC. [b] Reactions were performed in duplicate and results are reported as average of the two samples; n.d. = not detected; n.m. = not measured; n.c. = no conversion.

Table S6. One-pot oxidation/transamination of (*R*)-**5** (10 mM) catalyzed by ADHs in combination with one of two stereocomplementary ωTAs at different enzymes loading in $HCOONH_4$ buffer (pH 8.5, 1 M, 30 °C).

Entry	ADH [µM]	ωTA [µM]	Conv.[%]	7 [%]	*ee* 7 [%][b]	6 [%]
1	Aa-ADH [24]	Cv-ωTA [60]	48±12	31±10	>99 (*R*) [c]	17±2
2	Aa-ADH [70]	Cv-ωTA [35]	6±2	2±<1	n.m.	4±2
3	Aa-ADH [70]	Bm-ωTA [35]	71±<1	70±<1	>99 (*R*)[c]	1±<1
4	Aa-ADH [70]	Vf-ωTA[a]	n.c.	n.d.	n.m.	n.d.
5	Aa-ADH [50]	Ac-ωTA [50]	n.c.	n.d.	n.m.	n.d.
6	Aa-ADH [24]	At-ωTA [60]	99±1	96±1	>99 (*S*)[c]	4±1
7	Aa-ADH [70]	At-ωTA [35]	>99	97±<1	>99 (*S*)[c]	3±<1
8	Bs-BDHA [24]	At-ωTA [60]	>99	96±<1	>99 (*S*)[c]	4±<1
9	Bs-BDHA [70]	At-ωTA [35]	>99	95±<1	>99 (*S*)[c]	5±<1

[a] used as lyophilized *E. coli* whole cells (20 mg mL^{-1}); [b] analyzed by RP-HPLC (C18 HD column) after derivatization of the amino group with GITC. [c] Reactions were performed in duplicate and results are reported as average of the two samples; n.d. = not detected; n.m. = not measured; n.c. = no conversion.

6. One-pot concurrent disconnected two-step cascade for the conversion of either *rac*-5 or (*S*)-5 or (*R*)-5 into either (*S*)-9 or (*R*)-9 catalyzed by AcCO6 combined with Ch1-AmDH

In an Eppendorf tube (1.5 mL), $HCOONH_4$ buffer (0.5 mL, pH 8.5, 1 M), NAD^+ (1 mM), catalase (0.1 mg mL^{-1}) and purified Cb-FDH (10 µM) were added followed by purified AcCO6 (50 µM, otherwise stated in Table S7-8) and Ch1-AmDH (50 µM, otherwise stated in Table S7-8). *Rac*-**5** or (*R*)-**5** or (*S*)-**5** (10-20 mM) was added as last. The mixture was incubated at 30 °C, 170 rpm for 48 h on an orbital shaker and, after that, quenched with 10 M KOH (100 µL). The aqueous phase was saturated with solid NaCl and the organic compounds was extracted with EtOAc (1 x 500 µL). The organic layer was dried over $MgSO_4$ and analyzed by GC-FID to determine the conversion, while the enantiomeric excesses was analyzed by RP-HPLC after derivatization with a chiral reagent (GITC).

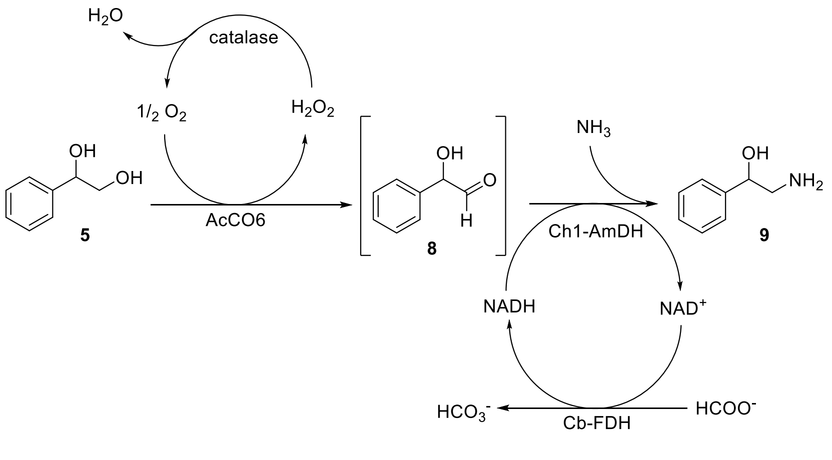

Figure S9. General scheme for the biocatalytic amination of **5** to **9**.

Table S7. Biocatalytic amination of *rac*- or (*S*)- or (*R*)-**5** (10-20 mM) into *rac*- or (*S*)- or (*R*)-**9** catalyzed by AcCO6 (50 µM) coupled with Ch1-AmDH (50 µM) in a one-pot cascade in $HCOONH_4$ buffer (pH 8.5, 1 M; 30 °C)

Entry	Substrate [mM]	Conv.[%]	9 [%]	Benzylamine (11) [%]	*ee* 9 [%][a]
1	*rac*-**5** [10]	>99	>99	n.d.	10 (*S*)
2	*rac*-**5** [20]	92±<1	90±<1	2±<1	10 (*S*)
3	(*S*)-**5** [10]	>99	98±<1	2±<1	>98 (*S*)
4	(*S*)-**5** [20]	96±1	88±1	8±<1	>98 (*S*)
5	(*R*)-**5** [10]	94±<1	92±<1	2±<1	>99 (*R*)
6	(*R*)-**5** [20]	78±1	72±1	6±<1	>99 (*R*)

[a] analyzed by RP-HPLC (C18 HD column) after derivatization of the amino group with GITC; reactions were performed in duplicate and results are reported as average of the two samples.

Table S8. Optimization of the biocatalytic amination of *rac*- or (*S*)- or (*R*)-**5** (10-30 mM) into *rac*- or (*S*)- or (*R*)-**9** catalyzed by AcCO6 (50 µM) coupled with Ch1-AmDH (50 µM) in a one-pot cascade in $HCOONH_4$ buffer (pH 8.5, 1 M; 30 °C) by varying substrate and enzyme loading

Entry	Sub. [mM]	AcCO6 [uM]	Ch1-AmDH [uM]	Conv. [%]	9 [%]	Benzylamine (11) [%]	*ee* 9 [%][b]
1[a]	(*S*)-**5** [20]	70	35	99±1	89±<1	11±1	>98 (*S*)
2	(*S*)-**5** [20]	24	60	81±4	77±3	4±1	n.m.
3	(*S*)-**5** [20]	10	50	43±<1	41±<1	2±<1	n.m.
4	(*R*)-**5** [10]	70	35	>99	98±<1	2±<1	>99 (*R*)
5	(*R*)-**5** [10]	24	60	77±<1	76±<1	1±<1	n.m.
6	(*R*)-**5** [10]	10	50	41±1	40±1	1±<1	n.m.
7	(*rac*)-**5** [30]	70	35	94±1	85±1	9±<1	38 (*S*)
8	(*rac*)-**5** [30]	24	60	62±1	60±1	2±<1	n.m.
9	(*rac*)-**5** [30]	10	50	31±<1	30±<1	1±<1	n.m.
10	(*rac*)-**5** [20]	70	35	99±1	96±<1	4±1	33 (*S*)
11	(*rac*)-**5** [20]	24	60	75±2	75±2	<1	n.m.
12	(*rac*)-**5** [20]	10	50	42±1	42±1	<1	n.m.

[a]Average of four samples; [b] analyzed by RP-HPLC (C18 HD column) after derivatization of the amino group with GITC; reactions were performed in duplicate and results are reported as average of the two samples.

7. Study on the formation of benzylamine (11) as side-product during the oxidation of 5

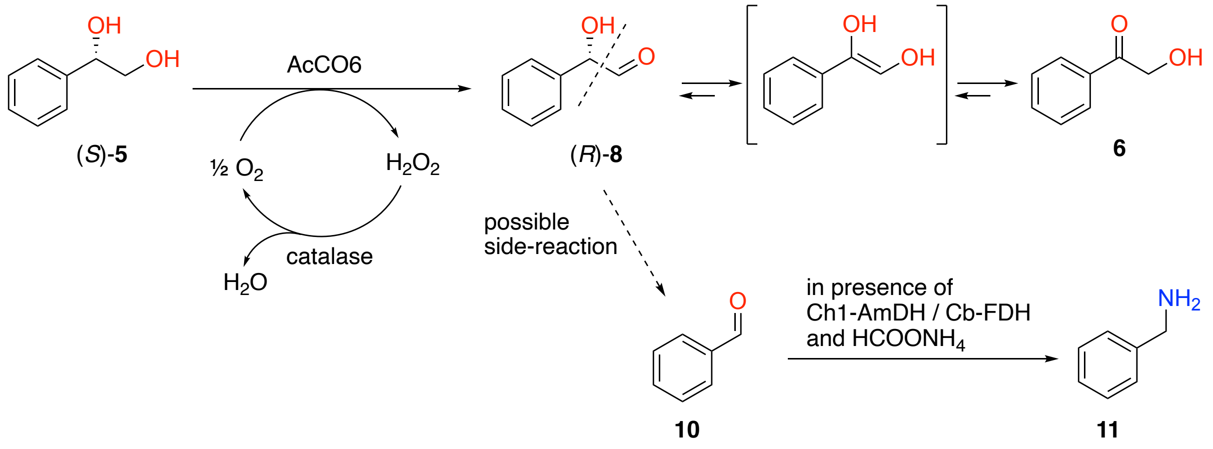

Figure S10. Enzymatic oxidation of (*S*)-**5** catalyzed by AcCO6 and possible side reactions leading to benzaldehyde (**10**) and benzylamine (**11**), the latter formed in presence of an amine dehydrogenase.

Experimental conditions

In an Eppendorf tube (2 mL), AcCO6 (50 µM) and catalase (0.1 mg mL^{-1}) were added in $HCOONH_4$ buffer (1 mL, pH 8.5, 1 M). (*S*)-**5** (20 mM) was added as last. The mixture was incubated at 30 °C, 170 rpm for 6 h or 48 h on an orbital shaker and, after that, quenched with 10 M KOH (100 µL). The aqueous phase was saturated with solid NaCl and the organic compounds was extracted with EtOAc (2 x 500 µL). The organic layer was dried over $MgSO_4$ and analyzed by GC-FID and GC-MS to determine any possible formation of benzaldehyde (**10**).

A negative control experiment was performed by incubating (*S*)-**5** (20 mM) in $HCOONH_4$ buffer (1 mL, pH 8.5, 1 M). Work-up was performed as described above.

Table S9. Study on the formation of benzaldehyde (**10**) from (*S*)-**5** (20 mM) in presence of AcCO6 (50 µM) and catalase (0.1 mg mL^{-1}) in $HCOONH_4$ buffer (pH 8.5, 1 M) at 30 °C.

Experiment	Time [h]	Conversion [%]
With AcCO6 and catalase	6	27
	48	32
Without AcCO6 and catalase	6	n.d.
	48	n.d.

n.d. = not detected.

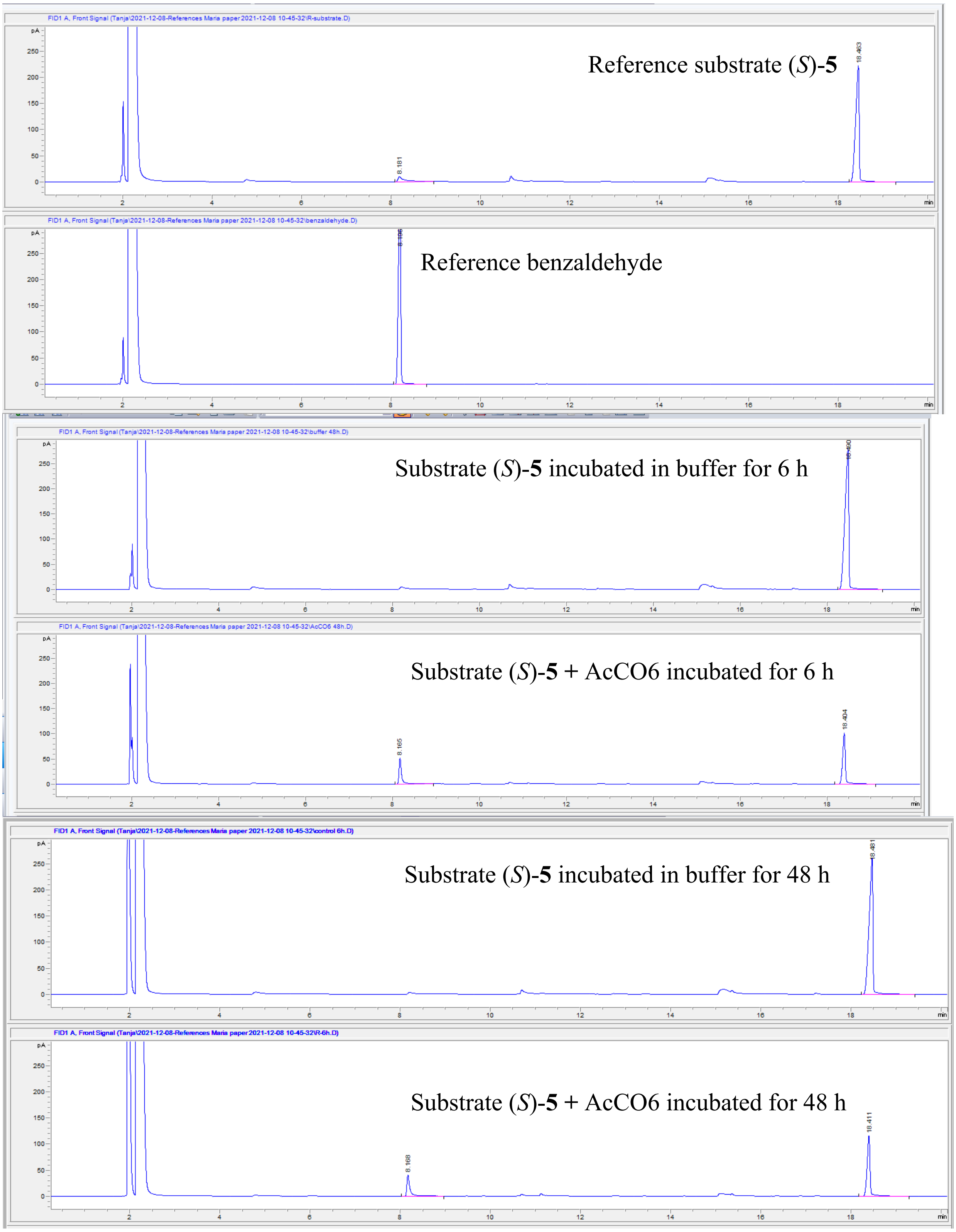

Figure S11. Progress of the reactions (at 6 h and 48 h) on (*S*)-**5** catalyzed by AcCO6/catalase in $HCOONH_4$ buffer (pH 8.5, 1 M) and negative control experiment.

8. Biocatalytic conversions of (*R*)-1-phenylethane-1,2-diol ((*R*)-5) into either (*S*)-2-phenylglycinol ((*S*)-7) or (*R*)-phenylethanolamine ((*R*)-9) at ca. 100 mg scale

The experimental procedures are reported in the main manuscript.

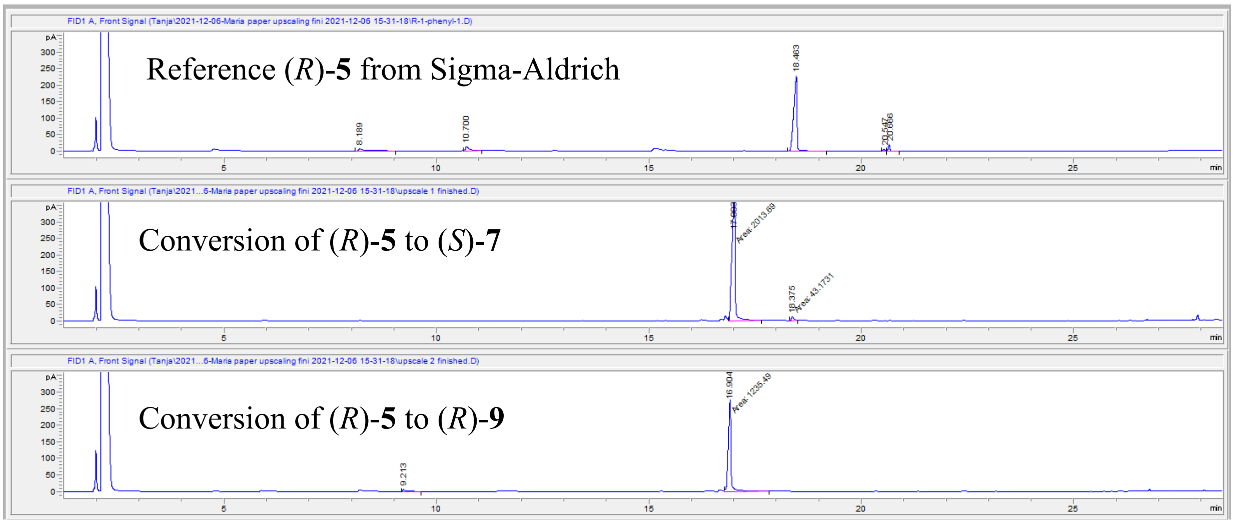

Figure S12. GC-chromatograms for the conversion of (*R*)-**5** into either (*S*)-**7** or (*R*)-**9** at ca. 100-mg scale; reference starting material and composition of the reaction mixtures after the biocatalytic reactions.

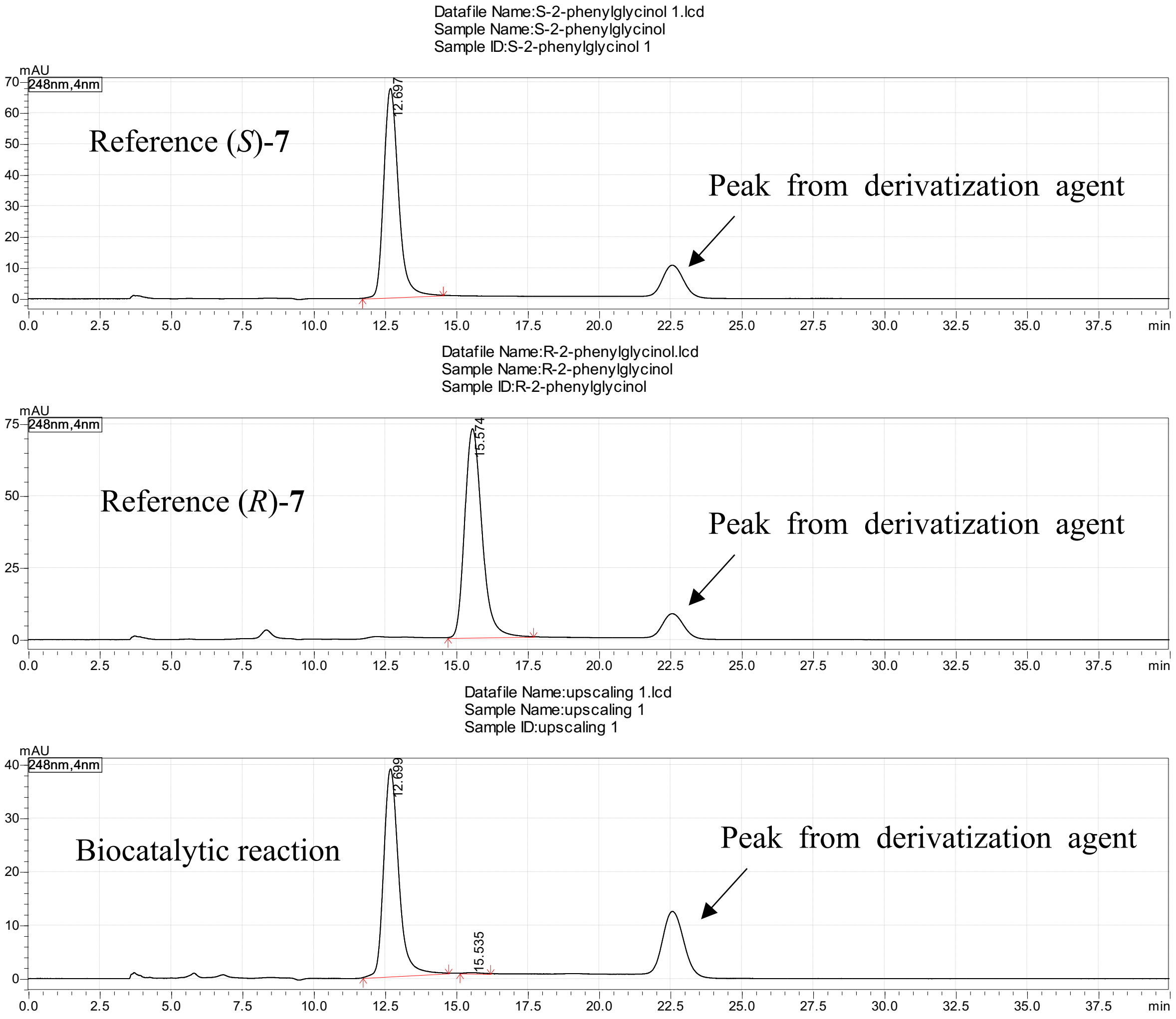

Figure S13. RP-HPLC chromatograms of (*S*)-**7** (obtained from the biocatalytic conversion of (*R*)-**5** at ca. 100-mg scale) after derivatization with GITC (for derivatization procedure see SI section S10). **Measured peaks area percentage:** t= 12.7 min 99.711% (main enantiomer); t= 15.5 min 0.289% (minor enantiomer).

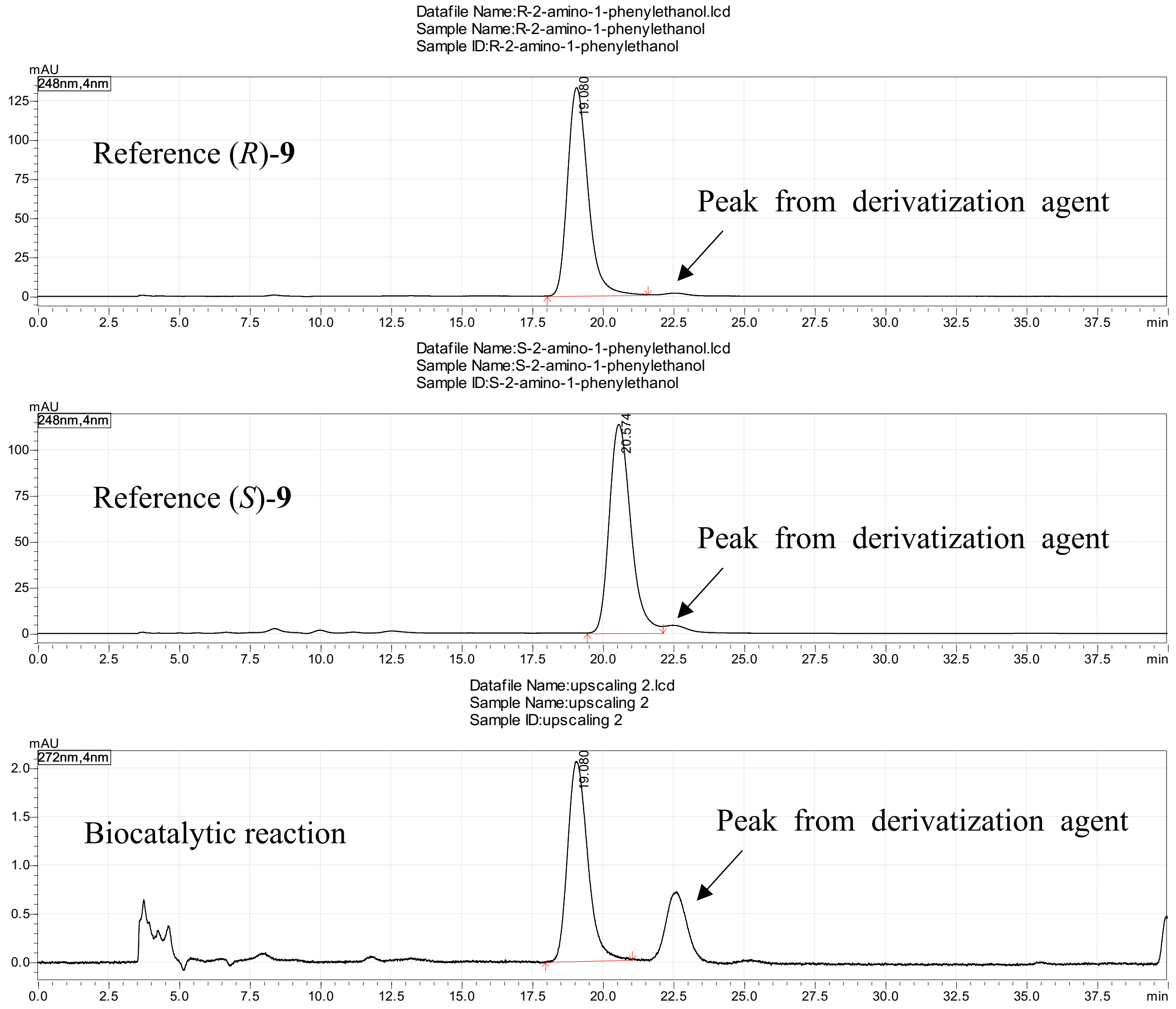

Figure S14. RP-HPLC chromatograms of (*R*)-**9** (obtained from the biocatalytic conversion of (*R*)-**5** at ca. 100-mg scale) after derivatization with GITC (for derivatization procedure see SI section S10). **Measured peaks area percentage:** t= 19.1 min 100% (main enantiomer); t= 20.6 min 0% (minor enantiomer).

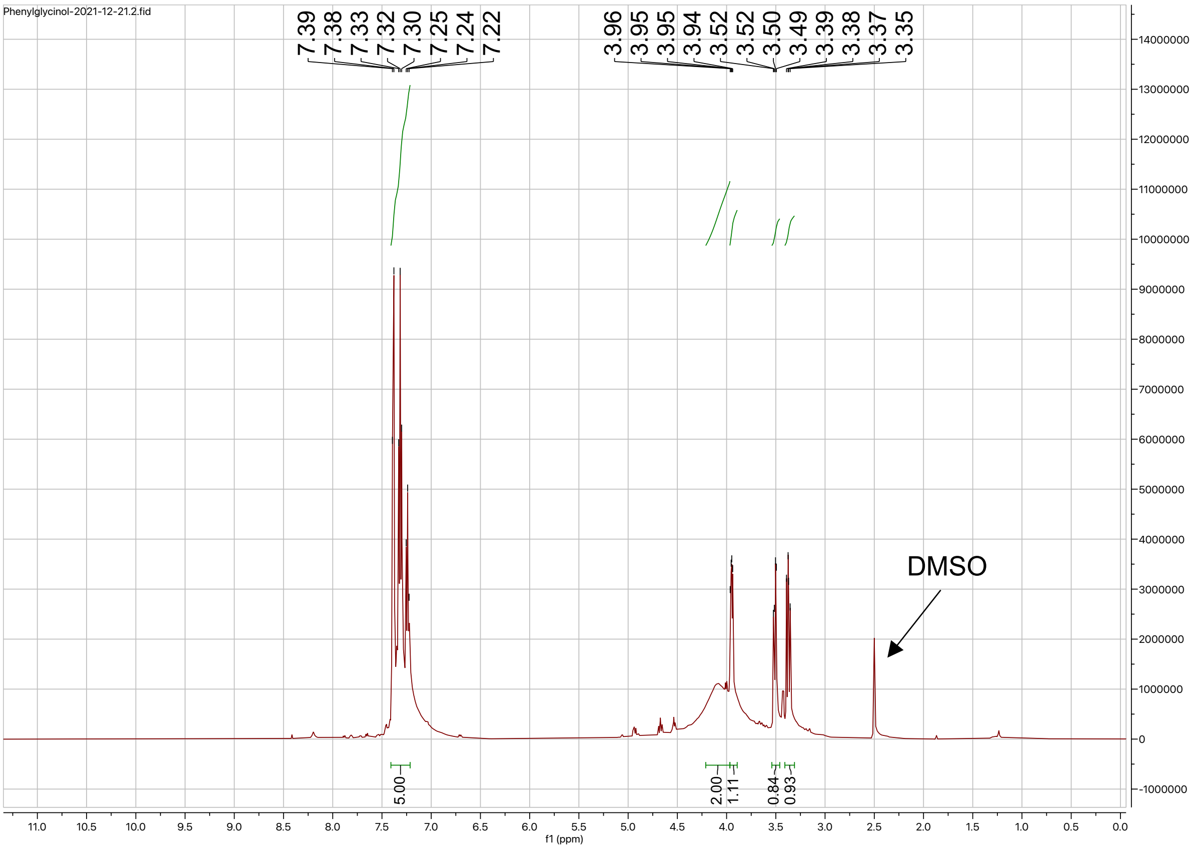

Figure S15. ^{1}H-NMR of (*S*)-**7** (obtained from the biocatalytic conversion of (*R*)-**5** at ca. 100-mg scale)

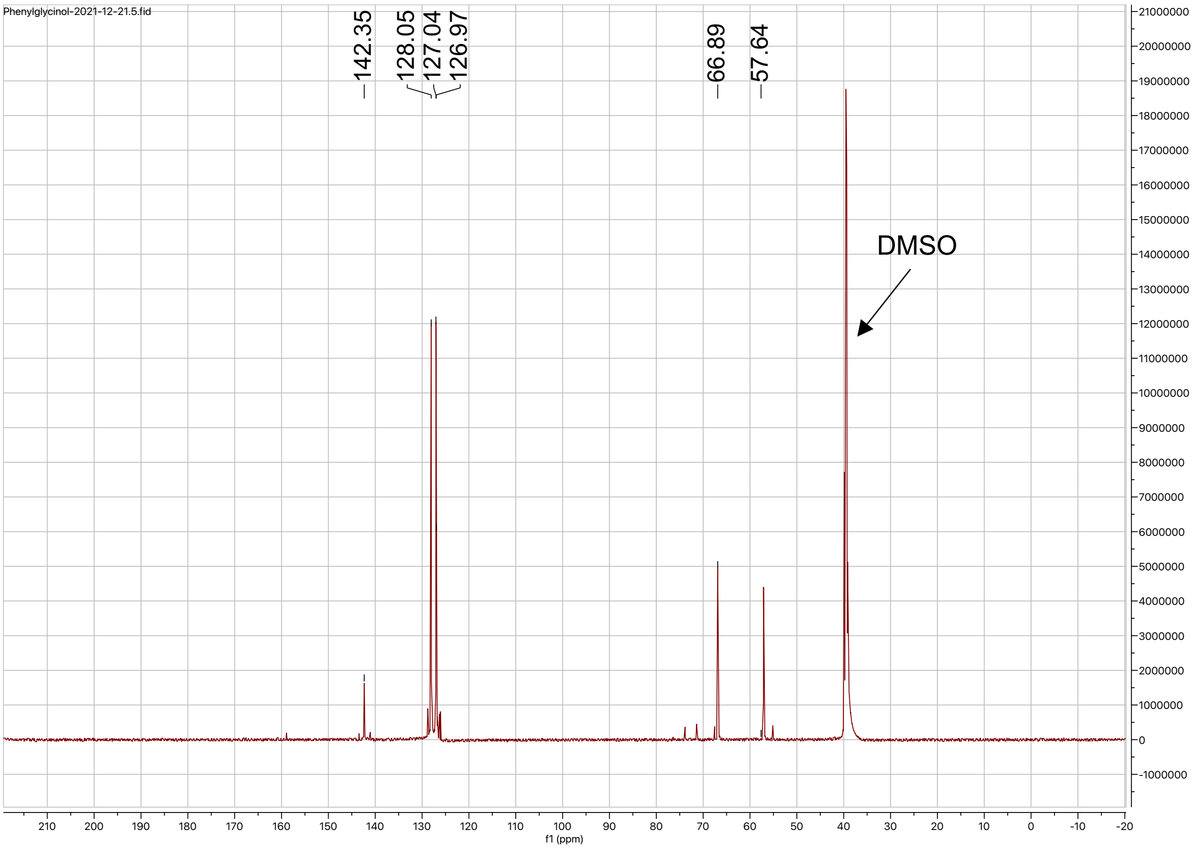

Figure S16. ^{13}C-NMR of (*S*)-**7** in DMSO-d6 (obtained from the biocatalytic conversion of (*R*)-**5** at ca. 100-mg scale).

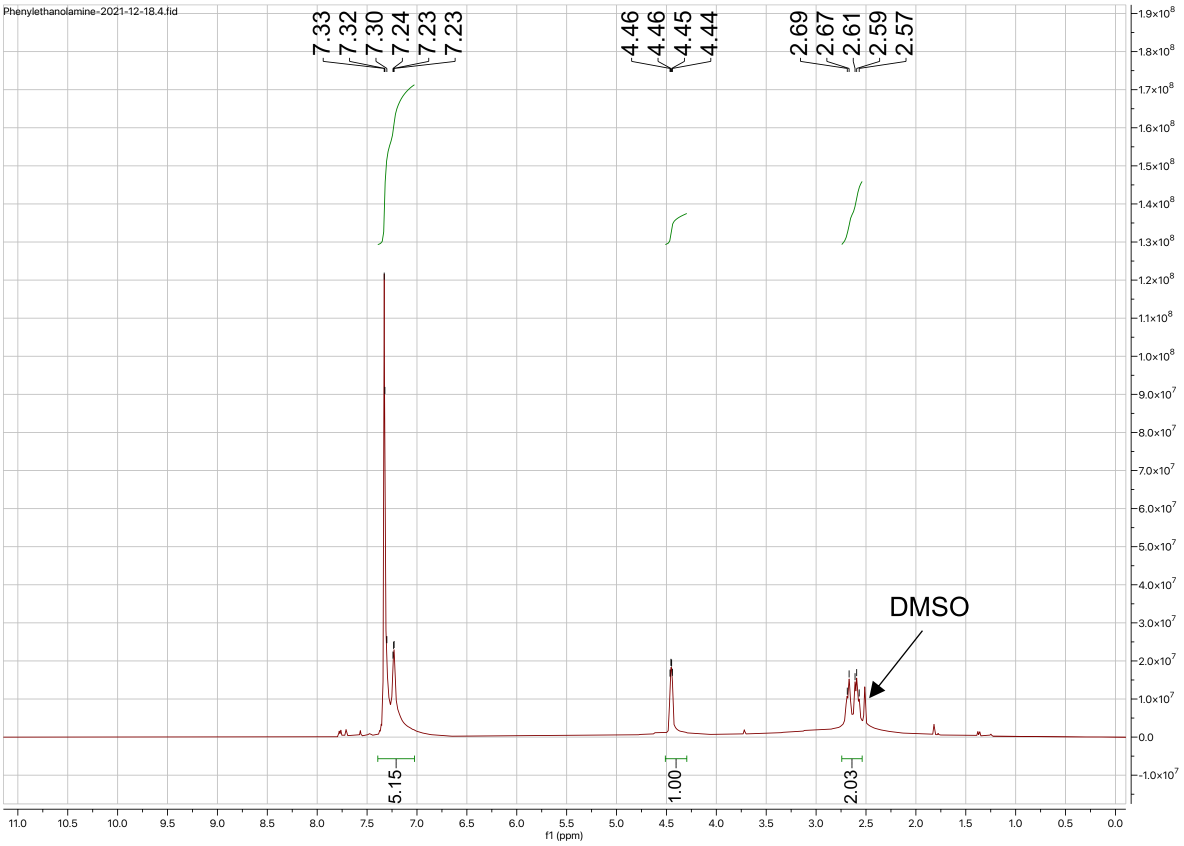

Figure S17. ^{1}H-NMR of (*R*)-**9** (obtained from the biocatalytic conversion of (*R*)-**5** at ca. 100-mg scale)

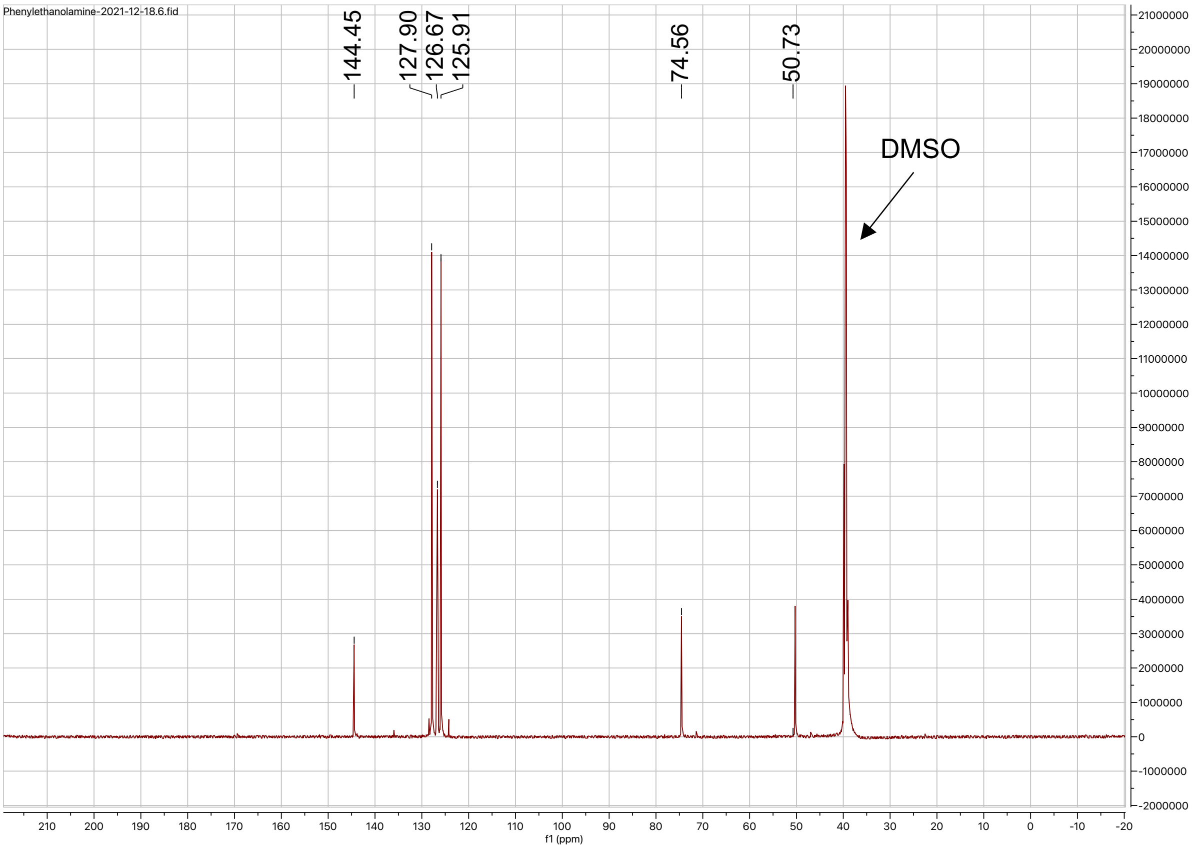

Figure S18. ^{13}C-NMR of (*R*)-**9** (obtained from the biocatalytic conversion of (*R*)-**5** at ca. 100-mg scale)

9. Representative GC-FID and HPLC chromatograms for the biocatalytic aminations of diols 5

- GC-FID chromatograms for the conversion of **3** into either (*R*)-**5** or (*S*)-**5** catalyzed by Fus-SMO/FDH combined with either St(R)-EH or Sp(S)-EH.

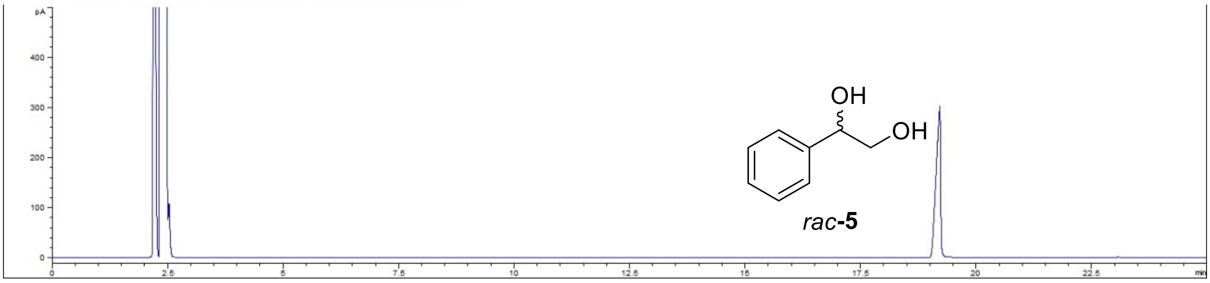

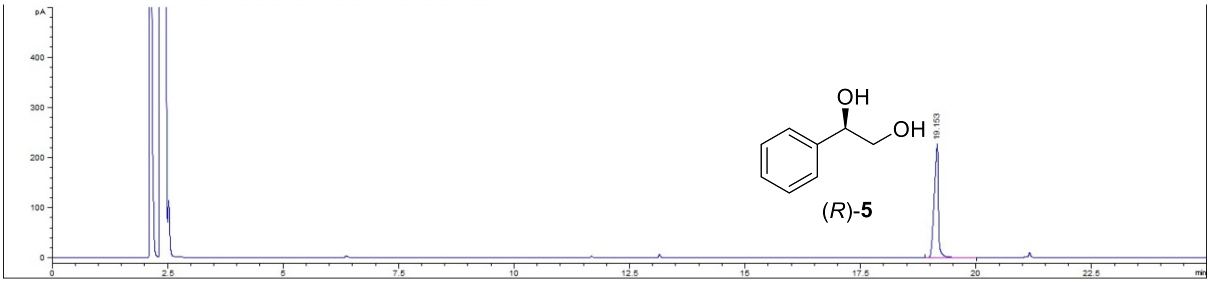

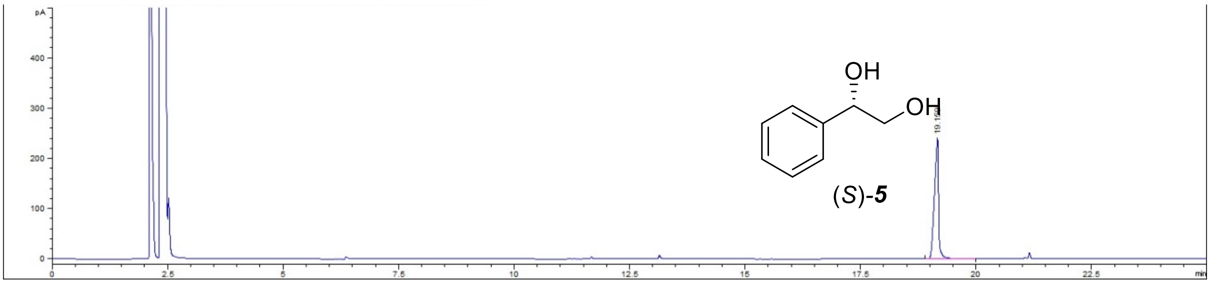

- Chiral NP-HPLC chromatograms for the determination of the enantiomeric excess of enzymatically synthesized (*R*)-**5** and (*S*)-**5**

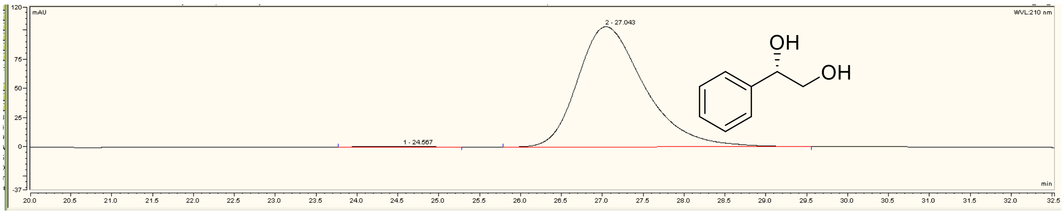

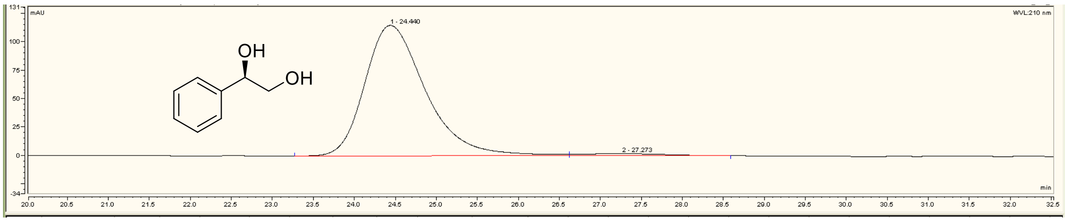

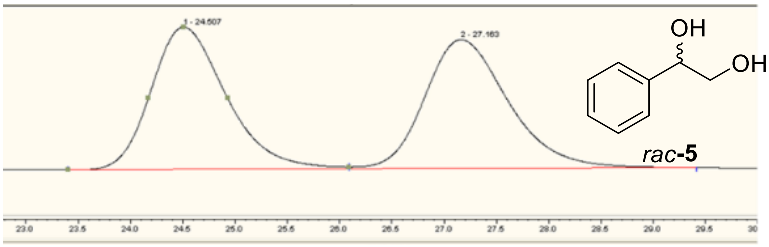

- GC-FID chromatograms for the conversion of (*R*)-**5** to (*S*)-**7** catalyzed by Aa-ADH combined with At(R)-ωTA.

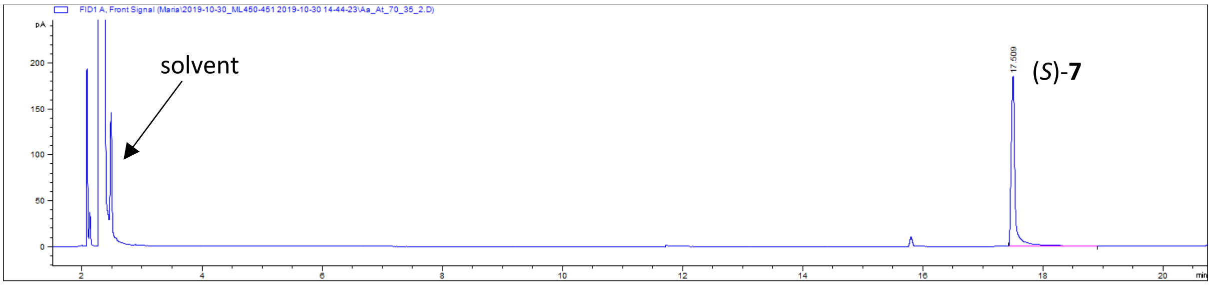

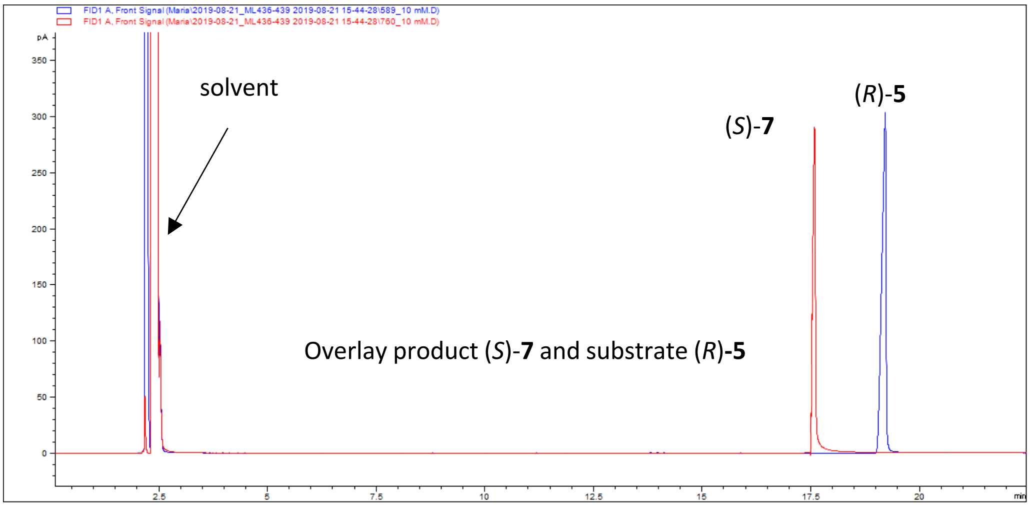

- RP-HPLC chromatograms for the determination of enantiomeric excess of the enzymatically synthesized (*S*)-**7**

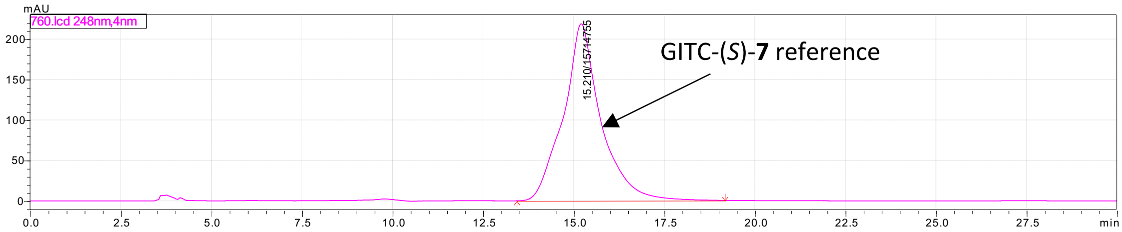

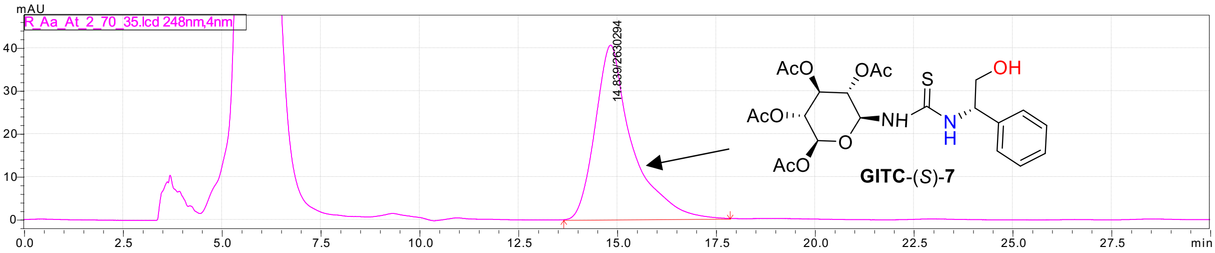

- GC-FID chromatograms for the conversion of (*R*)-**5** to (*R*)-**7** catalyzed by Aa-ADH combined with Bm(*S*)-ωTA

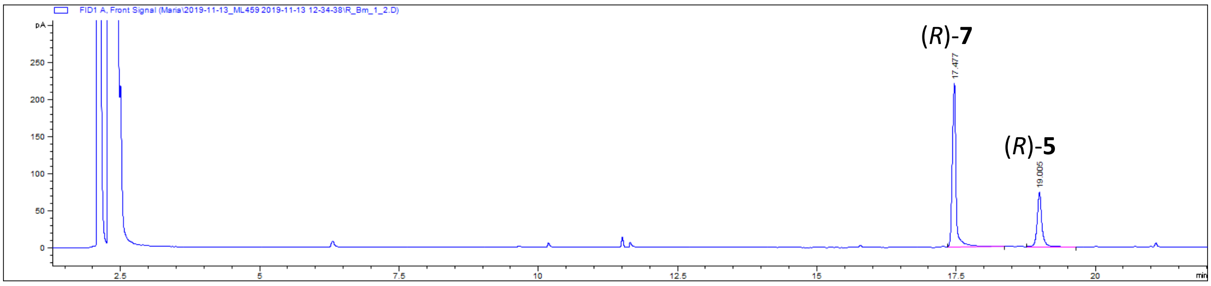

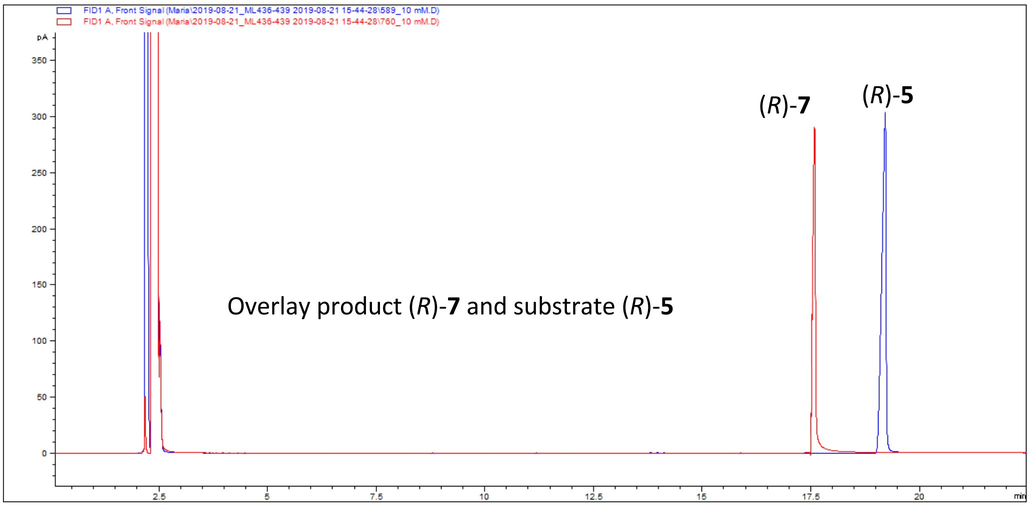

- RP-HPLC chromatograms for the determination of enantiomeric excess of the enzymatically synthesized (*R*)-**7**

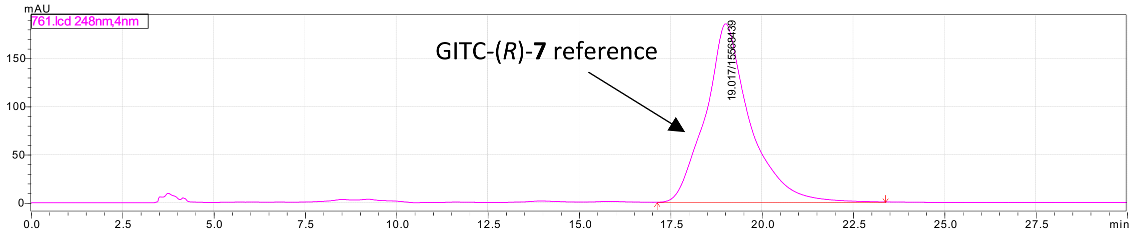

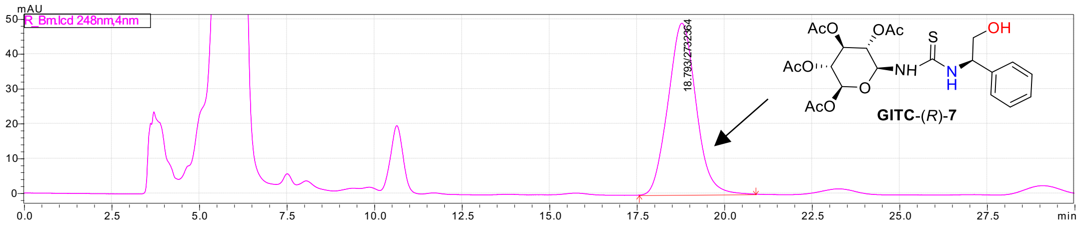

- GC-FID chromatograms for the conversion of (*S*)-**5** to (*S*)-**9** catalyzed by AcCO6 combined with Ch1-AmDH

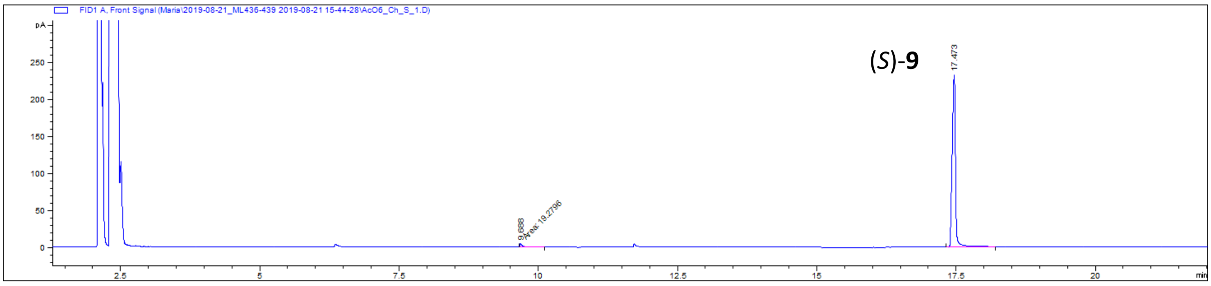

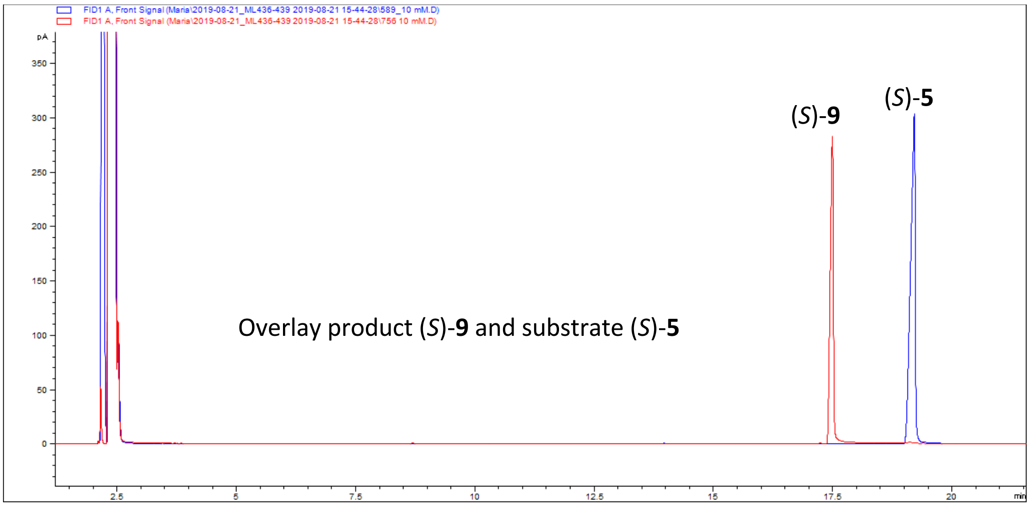

- RP-HPLC chromatograms for the determination of enantiomeric excess of the enzymatically synthesized (*S*)-**9**

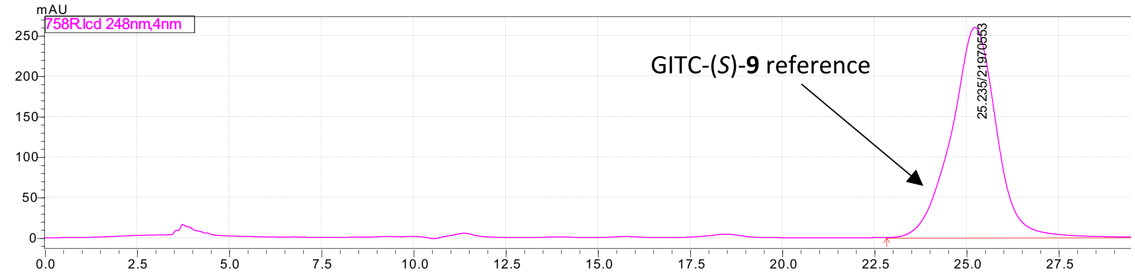

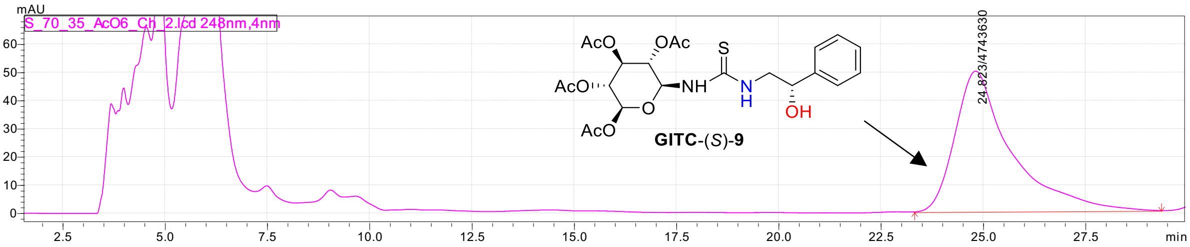

- GC-FID chromatograms for the conversion of (*R*)-**5** to (*R*)-**9** catalyzed by AcCO6 combined with Ch1-AmDH

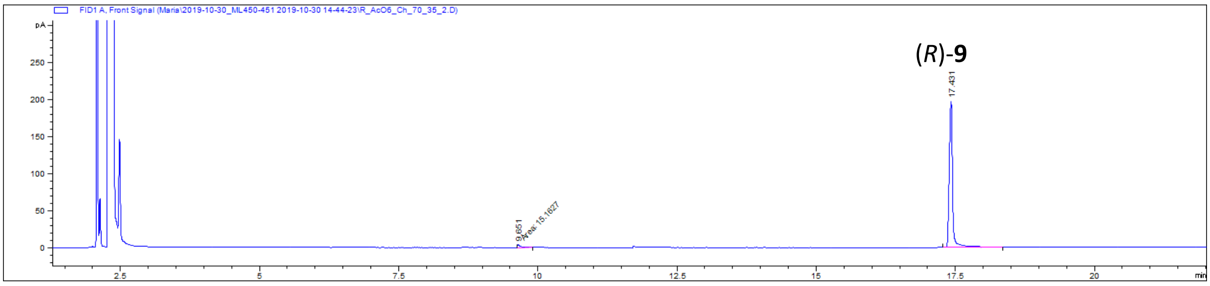

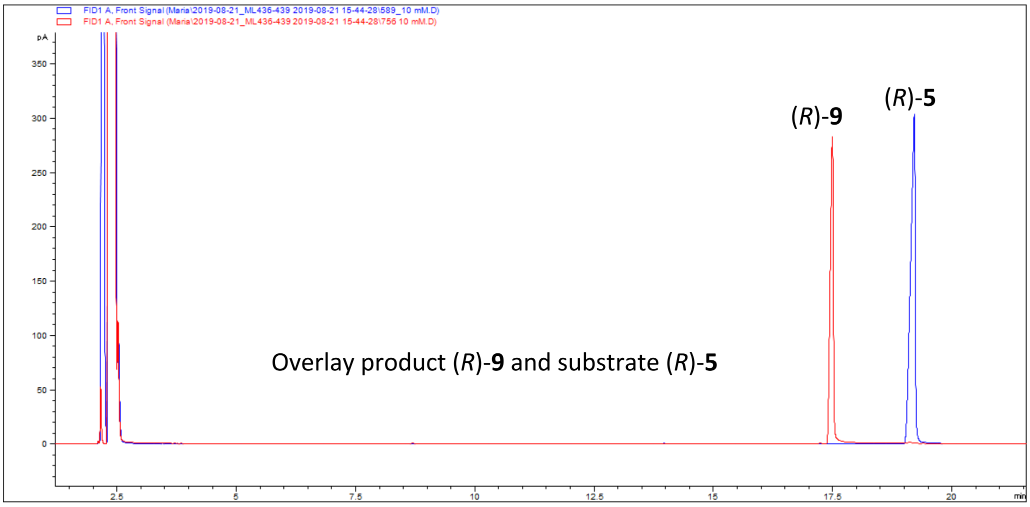

- RP-HPLC chromatograms for the determination of enantiomeric excess of the enzymatically synthesized (*R*)-**9**

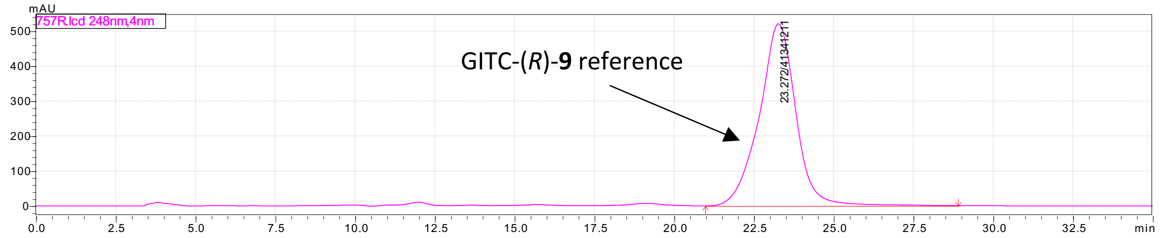

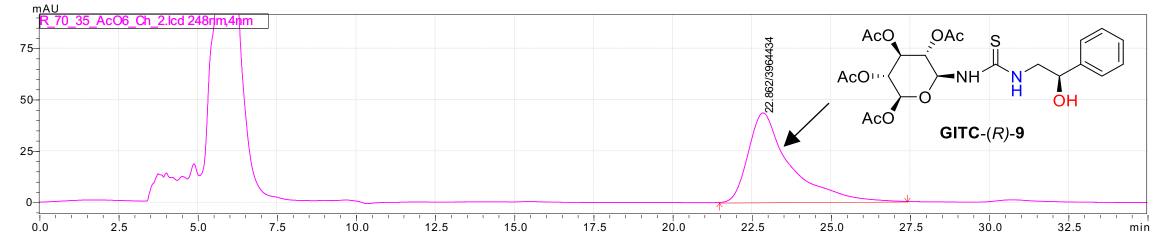

10. General derivatization procedure for the determination of the enantiomeric excess by RP-HPLC

The procedure was adapted from our previous protocol and other literature.[1, 36] The aqueous reaction mixture (20 µL) was dissolved in acetonitrile (180 µL) to yield a final concentration of 0.5 mM. Then, GITC (2,3,4,6-Tetra-O-acetyl-β-D-glucopyranosyl isothiocyanate) (1.5 mM) and Et_3N (1.5 mM) were added as a solution in acetonitrile (200 µL). The mixture was incubated at room temperature at 1000 rpm with an Eppendorf thermomixer for 35 min. Before injection into the RP-HPLC, the samples were centrifuged and filtered if required.

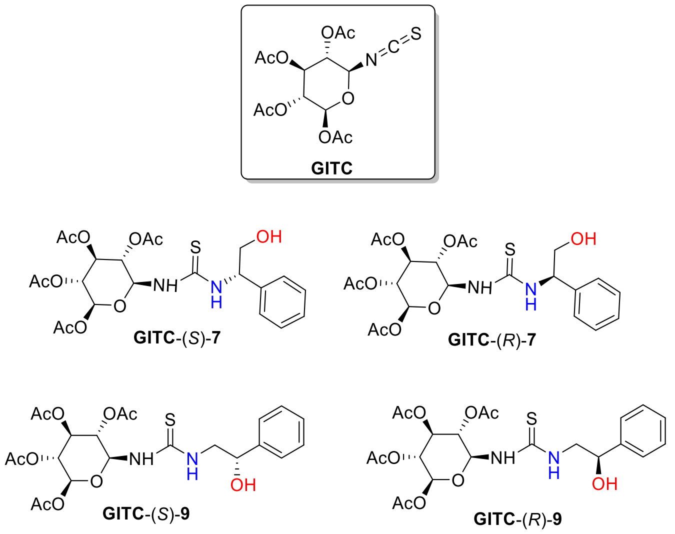

Figure S19. Structures of derivatized amino alcohols for ee determination

11. Analytical methods

__GC-FID method A__: Column Agilent DB-1701 (30 m, 250 µm, 0.25 µm); injector temperature 250 °C; constant pressure 14.50 psi; temperature program: 80 °C/hold 6.5 min; 160 °C/rate 10 °C min^{-1}/hold 5 min; 200 °C/rate 20 °C min^{-1}/hold 2 min; 280 °C/rate 20 °C min^{-1}/hold 1 min.

NP-HPLC method B: Column Chiralcel OD-H; Program: constant oven temperature 25 °C; constant pressure 14 bar; eluent composition: *n*-Hexane/Isopropanol 95:5, 1 mL min^{-1}; detection at 210 nm.

RP-HPLC method C: Column Shimpack C18-HD (0.46 cm x 25 cm); program: constant oven temperature 30 °C; eluent composition: isocratic; Eluent A: MeOH + 0.1% TFA, Eluent B: MilliQ + 0.1% TFA, Eluent A/Eluent, 50/50, v v^{-1}; flow rate: 1 mL min^{-1}, detection at 248 nm.

RP-HPLC method D: Column: Nucleosil C_{18} HD (0.46 cm x 25 cm); program: constant oven temperature 30 °C; eluent composition: isocratic; Eluent A: MeOH + 0.1% TFA, Eluent B: MilliQ + 0.1% TFA, Eluent A/Eluent, 50/50, v v^{-1};; flow rate: 0.7 mL min^{-1}, detection at 248 nm.

Table S10. Retention times of compounds used or synthesized in this study and associated analytical methods.

Compound	Retention time [min]	Analytical method
L-Phenylalanine (**1**)	2.4	C (achiral column)
Cinnamic acid (**2**)	9.2	C (achiral column)
Styrene (**3**)	27.9	C (achiral column)
	4.4	A (achiral column)
(*S*)-styrene oxide, ((*S*)-**4**)	10.3	A (achiral column)
(*S*)-1-phenylethane-1,2-diol, ((*S*)-**5**)	19.1	A (achiral column)
	27.1	B (chiral column)
(*R*)-1-phenylethane-1,2-diol, (*R*)-**5**	19.1	A (achiral column)
	24.5	B (chiral column)
2-hydroxy-acetophenone (**6**)	15.8	A (achiral column)
(*S*)-2-phenylglycinol, ((*S*)-**7**)[a]	17.5	A (achiral column)
	15.0	D (achiral column)
(*R*)-2-phenylglycinol, ((*R*)-**7**)[a]	17.5	A (achiral column)
	19.0	D (achiral column)
(*S*)-phenylethanolamine, ((*S*)-**9**)[a]	17.4	A (achiral column)
	25.1	D (achiral column)
(*R*)-phenylethanolamine, ((*R*)-**9**)[a]	17.4	A (achiral column)
	23.0	D (achiral column)

[a] after derivatization with GITC

12. References

1. Corrado, M. L.; Knaus, T.; Mutti, F. G., Regio- and stereoselective multi-enzymatic aminohydroxylation of β-methylstyrene using dioxygen, ammonia and formate. *Green Chem.* **2019,** *21*, 6246-6251.
2. Bohmer, W.; Knaus, T.; Volkov, A.; Slot, T. K.; Shiju, N. R.; Engelmark Cassimjee, K.; Mutti, F. G., Highly efficient production of chiral amines in batch and continuous flow by immobilized omega-transaminases on controlled porosity glass metal-ion affinity carrier. *J. Biotechnol.* **2019,** *291*, 52-60.
3. Mertens, M. A. S.; Sauer, D. F.; Markel, U.; Schiffels, J.; Okuda, J.; Schwaneberg, U., Chemoenzymatic cascade for stilbene production from cinnamic acid catalyzed by ferulic acid decarboxylase and an artificial metathease. *Catal. Sci. Technol.* **2019,** *9*, 5572-5576.
4. Heath, R. S.; Birmingham, W. R.; Thompson, M. P.; Taglieber, A.; Daviet, L.; Turner, N. J., An Engineered Alcohol Oxidase for the Oxidation of Primary Alcohols. *ChemBioChem* **2019,** *20*, 276-281.
5. Pilbak, S.; Farkas, O.; Poppe, L., Mechanism of the tyrosine ammonia lyase reaction-tandem nucleophilic and electrophilic enhancement by a proton transfer. *Chemistry* **2012,** *18*, 7793-7802.
6. Busto, E.; Simon, R. C.; Kroutil, W., Vinylation of Unprotected Phenols Using a Biocatalytic System. *Angew. Chem. Int. Ed.* **2015,** *54*, 10899-902.
7. Corrado, M. L.; Knaus, T.; Mutti, F. G., A Chimeric Styrene Monooxygenase with Increased Efficiency in Asymmetric Biocatalytic Epoxidation. *ChemBioChem* **2018,** *19*, 679-686.

8. Wu, S.; Chen, Y.; Xu, Y.; Li, A.; Xu, Q.; Glieder, A.; Li, Z., Enantioselective trans-Dihydroxylation of Aryl Olefins by Cascade Biocatalysis with Recombinant Escherichia coli Coexpressing Monooxygenase and Epoxide Hydrolase. *ACS Catal.* **2014,** *4*, 409-420.
9. Lavandera, I.; Kern, A.; Ferreira-Silva, B.; Glieder, A.; de Wildeman, S.; Kroutil, W., Stereoselective bioreduction of bulky-bulky ketones by a novel ADH from Ralstonia sp. *J Org Chem* **2008,** *73*, 6003-6005.
10. Hoffken, H. W.; Duong, M.; Friedrich, T.; Breuer, M.; Hauer, B.; Reinhardt, R.; Rabus, R.; Heider, J., Crystal structure and enzyme kinetics of the (S)-specific 1-phenylethanol dehydrogenase of the denitrifying bacterium strain EbN1. *Biochemistry* **2006,** *45*, 82-93.
11. Bohmer, W.; Knaus, T.; Mutti, F. G., Hydrogen-Borrowing Alcohol Bioamination with Coimmobilized Dehydrogenases. *ChemCatChem* **2018,** *10*, 731-735.
12. Lavandera, I.; Holler, B.; Kern, A.; Ellmer, U.; Glieder, A.; de Wildeman, S.; Kroutil, W., Asymmetric anti-Prelog reduction of ketones catalysed by Paracoccus pantotrophus and Comamonas sp cells via hydrogen transfer. *Tetrahedron: Asymmetry* **2008,** *19*, 1954-1958.
13. Lavandera, I.; Kern, A.; Resch, V.; Ferreira-Silva, B.; Glieder, A.; Fabian, W. M.; de Wildeman, S.; Kroutil, W., One-way biohydrogen transfer for oxidation of sec-alcohols. *Org. Lett.* **2008,** *10*, 2155-2158.
14. Knaus, T.; Cariati, L.; Masman, M. F.; Mutti, F. G., In vitro biocatalytic pathway design: orthogonal network for the quantitative and stereospecific amination of alcohols. *Org Biomol Chem* **2017,** *15*, 8313-8325.
15. Niefind, K.; Muller, J.; Riebel, B.; Hummel, W.; Schomburg, D., The crystal structure of R-specific alcohol dehydrogenase from Lactobacillus brevis suggests the structural basis of its metal dependency. *J. Mol. Biol.* **2003,** *327*, 317-828.
16. Schlieben, N. H.; Niefind, K.; Muller, J.; Riebel, B.; Hummel, W.; Schomburg, D., Atomic resolution structures of R-specific alcohol dehydrogenase from Lactobacillus brevis provide the structural bases of its substrate and cosubstrate specificity. *J. Mol. Biol.* **2005,** *349*, 801-813.
17. Mutti, F. G.; Knaus, T.; Scrutton, N. S.; Breuer, M.; Turner, N. J., Conversion of alcohols to enantiopure amines through dual-enzyme hydrogen-borrowing cascades. *Science* **2015,** *349*, 1525-1529.
18. Inoue, K.; Makino, Y.; Itoh, N., Purification and characterization of a novel alcohol dehydrogenase from Leifsonia sp. strain S749: a promising biocatalyst for an asymmetric hydrogen transfer bioreduction. *Appl. Environ. Microbiol.* **2005,** *71*, 3633-3641.
19. Zhang, J.; Xu, T.; Li, Z., Enantioselective Biooxidation of Racemic trans-Cyclic Vicinal Diols: One-Pot Synthesis of Both Enantiopure (S,S)-Cyclic Vicinal Diols and (R)-α-Hydroxy Ketones. *Adv. Synth. Catal.* **2013,** *355*, 3147-3153.
20. Zhang, J.; Wu, S.; Wu, J.; Li, Z., Enantioselective Cascade Biocatalysis via Epoxide Hydrolysis and Alcohol Oxidation: One-Pot Synthesis of (R)-α-Hydroxy Ketones from Meso- or Racemic Epoxides. *ACS Catal.* **2014,** *5*, 51-58.
21. Bommarius, B. R.; Schurmann, M.; Bommarius, A. S., A novel chimeric amine dehydrogenase shows altered substrate specificity compared to its parent enzymes. *Chem. Commun.* **2014,** *50*, 14953-14955.
22. Ye, L. J.; Toh, H. H.; Yang, Y.; Adams, J. P.; Snajdrova, R.; Li, Z., Engineering of Amine Dehydrogenase for Asymmetric Reductive Amination of Ketone by Evolving Rhodococcus Phenylalanine Dehydrogenase. *ACS Catal.* **2015,** *5*, 1119-1122.
23. Knaus, T.; Bohmer, W.; Mutti, F. G., Amine dehydrogenases: efficient biocatalysts for the reductive amination of carbonyl compounds. *Green Chem.* **2017,** *19*, 453-463.
24. Tseliou, V.; Knaus, T.; Masman, M. F.; Corrado, M. L.; Mutti, F. G., Generation of amine dehydrogenases with increased catalytic performance and substrate scope from epsilon-deaminating L-Lysine dehydrogenase. *Nat. Commun.* **2019,** *10*, 3717.
25. Morokutti, A.; Lyskowski, A.; Sollner, S.; Pointner, E.; Fitzpatrick, T. B.; Kratky, C.; Gruber, K.; Macheroux, P., Structure and function of YcnD from Bacillus subtilis, a flavin-containing oxidoreductase. *Biochemistry* **2005,** *44*, 13724-13733.
26. Knaus, T.; Tseliou, V.; Humphreys, L. D.; Scrutton, N. S.; Mutti, F. G., A biocatalytic method for the chemoselective aerobic oxidation of aldehydes to carboxylic acids. *Green Chem.* **2018,** *20*, 3931-3943.
27. Matsumoto, J.; Higuchi, M.; Shimada, M.; Yamamoto, Y.; Kamio, Y., Molecular cloning and sequence analysis of the gene encoding the H2O-forming NADH oxidase from Streptococcus mutans. *Biosci Biotechnol Biochem* **1996,** *60*, 39-43.
28. Ohashima, T.; Soda, K., Purification and properties of alanine dehydrogenase from Bacillus sphaericus. *Eur. J. Biochem.* **1979,** *100*, 29-30.
29. Lyskowski, A.; Gruber, C.; Steinkellner, G.; Schurmann, M.; Schwab, H.; Gruber, K.; Steiner, K., Crystal structure of an (R)-selective omega-transaminase from Aspergillus terreus. *PLoS One* **2014,** *9*, e87350.

30. Mutti, F. G.; Fuchs, C. S.; Pressnitz, D.; Sattler, J. H.; Kroutil, W., Stereoselectivity of Four (R)-Selective Transaminases for the Asymmetric Amination of Ketones. *Adv. Synth. Catal.* **2011,** *353*, 3227-3233.
31. Kaulmann, U.; Smithies, K.; Smith, M. E. B.; Hailes, H. C.; Ward, J. M., Substrate spectrum of ω-transaminase from Chromobacterium violaceum DSM30191 and its potential for biocatalysis. *Enzyme Microb. Technol.* **2007,** *41*, 628-637.
32. Hanson, R. L.; Davis, B. L.; Chen, Y.; Goldberg, S. L.; Parker, W. L.; Tully, T. P.; Montana, M. A.; Patel, R. N., Preparation of (R)-Amines from Racemic Amines with an (S)-Amine Transaminase fromBacillus megaterium. *Adv. Synth. Catal.* **2008,** *350*, 1367-1375.
33. van Oosterwijk, N.; Willies, S.; Hekelaar, J.; Terwisscha van Scheltinga, A. C.; Turner, N. J.; Dijkstra, B. W., Structural Basis of the Substrate Range and Enantioselectivity of Two (S)-Selective omega-Transaminases. *Biochemistry* **2016,** *55*, 4422-4431.
34. Mutti, F. G.; Fuchs, C. S.; Pressnitz, D.; Turrini, N. G.; Sattler, J. H.; Lerchner, A.; Skerra, A.; Kroutil, W., Amination of Ketones by Employing Two New (S)-Selective ω-Transaminases and the His-Tagged ω-TA from Vibrio fluvialis. *Eur. J. Org. Chem.* **2012,** *2012*, 1003-1007.
35. Shin, J. S.; Yun, H.; Jang, J. W.; Park, I.; Kim, B. G., Purification, characterization, and molecular cloning of a novel amine:pyruvate transaminase from Vibrio fluvialis JS17. *Appl. Microbiol. Biotechnol.* **2003,** *61*, 463-471.
36. Malik, M. S.; Park, E.-S.; Shin, J.-S., ω-Transaminase-catalyzed kinetic resolution of chiral amines using l-threonine as an amino acceptor precursor. *Green Chem.* **2012,** *14*, 2137.
